# Informing the Physical Activity Evaluation Framework: A Scoping Review of Reviews

**DOI:** 10.1177/08901171211050059

**Published:** 2021-12-06

**Authors:** Leanne Kosowan, Stephen Shannon, Janet Rothney, Gayle Halas, Jennifer Enns, Maxine Holmqvist, Pamela Wener, Leah Goertzen, Alan Katz

**Affiliations:** 1 Rady Faculty of Health Sciences, 8664 University of Manitoba, Winnipeg, MB, Canada; 2 Neil John Maclean Health Sciences Library, 8664 University of Manitoba, Winnipeg, MB, Canada.; 3 Manitoba Centre for Health Policy, 8664 University of Manitoba, Winnipeg, MB, Canada; 4 Department of Clinical Health Psychology, 423134 University of Manitoba Faculty of Health Sciences, Winnipeg, MB, Canada; 5 Department of Occupational Therapy, 423134 University of Manitoba Faculty of Health Sciences, Winnipeg, MB, Canada

**Keywords:** Primary prevention, physical activity, evaluation, health promotion, scoping review

## Abstract

**Objective:**

Robust program evaluations can identify effective promotion strategies. This scoping review aimed to analyze review articles (including systematic reviews, meta-analysis, meta-synthesis, scoping review, narrative review, rapid review, critical review, and integrative reviews) to systematically map and describe physical activity program evaluations published between January 2014 and July 2020 to summarize key characteristics of the published literature and suggest opportunities to strengthen current evaluations.

**Data Source:**

We conducted a systematic search of the following databases: Medline, Scopus, Sportdiscus, Eric, PsycInfo, and CINAHL.

**Inclusion/Exclusion Criteria:**

Abstracts were screened for inclusion based on the following criteria: review article, English language, human subjects, primary prevention focus, physical activity evaluation, and evaluations conducted in North America.

**Extraction:**

Our initial search yielded 3193 articles; 211 review articles met the inclusion criteria.

**Synthesis:**

We describe review characteristics, evaluation measures, and “good practice characteristics” to inform evaluation strategies.

**Results:**

Many reviews (72%) did not assess or describe the use of an evaluation framework or theory in the primary articles that they reviewed. Among those that did, there was significant variability in terminology making comparisons difficult. Process indicators were more common than outcome indicators (63.5% vs 46.0%). There is a lack of attention to participant characteristics with 29.4% capturing participant characteristics such as race, income, and neighborhood. Negative consequences from program participation and program efficiency were infrequently considered (9.3% and 13.7%).

**Conclusion:**

Contextual factors, negative outcomes, the use of evaluation frameworks, and measures of program sustainability would strengthen evaluations and provide an evidence-base for physical activity programming, policy, and funding.

## Objective

Physical activity is an important component of health promotion and primary prevention of chronic disease, disability, and injury^1–6^ Physical inactivity is an increasing global concern. It is the fourth leading risk factor contributing to mortality and is associated with cardiovascular disease, diabetes, cancer, and mental illness.^6-8^ Despite the development of strategies to address physical activity participation, the majority of the North American population does not reach suggested levels of physical activity.^3^ The World Health Organization (WHO)^6,9^ has defined the recommended level of physical activity for adults as:

- 150–300 minutes of moderate-intensity aerobic physical activity per week or
- At least 75–150 minutes of vigorous intensity aerobic physical activity per week or
- An equivalent combination of moderate- and vigorous-intensity activity throughout the week

Additionally, WHO^6^ suggests that children and youth should obtain 60 minutes of moderate-to-vigorous intensity physical activity per day. Only 18% of Canadian adults and 9.5% of Canadian children and youth are meeting physical activity guidelines.^3^

A program evaluation is “the systemic collection of information about the activities, characteristics and outcomes of a program to make judgements about the program, improve program effectiveness, and/or inform decisions about future program development.”^10^ Evaluations are important tools for program improvement while also exerting influence on policy and funding streams, building community capacity, and facilitating information sharing between communities and programs.^11,12^ As described by US Department of Health and Human Services (HHS),^12^ the type of evaluation used is dependent upon the purpose of the evaluation and when it is conducted within the program’s life cycle. Implementation/process evaluations assess the inputs and activities of a program (e.g., is the program being delivered as planned, what are the external influences, and is the program within time and resource capacity). Effectiveness/outcome evaluations measure the short-term, intermediate, or long-term effect(s) of the program (e.g., what was accomplished, is the program effective, and were there any unintended effects).^11,12^

There are national and global frameworks to support the development of physical activity initiatives and guide their evaluation. In 2006, the WHO developed the *Global Strategy on Diet, Physical Activity, and Health: A Framework to Monitor and Evaluate Implementation*.^13^ The objective of this strategic approach was to provide a framework and indicators that could be used in a physical activity program evaluation.^13^ Additionally, in 2011 the USA Centre for Disease Control and Prevention released a guide for evaluation in public health.^12^ In 2012, stakeholders of the pan-Canadian physical activity collaboration developed Active Canada 20/20, which provides a local, regional, provincial/territorial, and national framework for physical activity promotion.^14^ Active Canada 20/20 advocates for the adoption of population-based strategies, with specific attention to population sub-groups facing the greatest barriers to physical activity.^15^ This approach is supported by evidence that program participation and rates of physical activity are impacted by characteristics such as race, income, migrant status, and neighborhood factors.^5,11,13-16^ The first research action of the European Union’s Joint Programming initiative, the Determinants of Diet and Physical Activity (DEDIPAC) Knowledge Hub, conducted an umbrella review in 2015 to identify “Good Practice Characteristics” to assist in monitoring and evaluating interventions and policies that promote a healthy diet, increase participation in physical activity, and reduce sedentary behaviors.^17^ The Good Practice Characteristics addressed costs, outcomes, measurements, and process evaluation aspects.^17^ Together WHO, HHS, Active Canada 20/20, and DEDIPAC suggest a strategy to meet the diversity and complexity required to achieve sustainable behavior change as reflected in the Public Health Agency of Canada vision.^3^

To further this work, Kosowan et al^18^ assessed strengths, challenges, and opportunities in currently implemented physical activity strategies. One of the challenges that emerged was the need for guidance to develop and resource program evaluations that can inform physical activity strategies by highlighting current approaches as well as gaps and areas for improvement.^18^ This program evaluation challenge highlights several potentially relevant frameworks, indicators, and tools but also questions—to what extent are these frameworks and best practices reflected in published program evaluations? We therefore conducted this scoping review to systematically map and describe evaluations of physical activity programs in North America.

## Aim

This review aimed to analyze review articles to systematically map and describe physical activity program evaluations published between January 2014 and July 2020 to summarize key characteristics of the published literature and suggest opportunities to strengthen current evaluations. This review identified the presence of key characteristics outlined in national, Active Canada 20/20,^14,15^ and international, WHO,^6,13^ program evaluation frameworks. We targeted reviews conducted between 2014 and 2020, following the release of tools aimed at tailoring evaluations frameworks to the local context.^15^ In 2014, a national summit in Canada created, “Pathways to Wellbeing Framework for Recreation in Canada,” producing a plan and commitment to action developed by physical activity stakeholders to address inactivity by 2020.^15^ This Canadian plan references a similar plan developed for the United States of America,^19^ and was informed by international strategies to address physical inactivity.^13^ We provide direction for future evaluations by highlighting gaps in the literature and opportunities to strengthen current approaches to physical activity evaluation and monitoring.

A scoping review provides an overview of extent, range, and nature of research activity available on a given topic.^20^ Scoping reviews are well suited for understanding gaps in the research area of interest. Scoping reviews can range from a rapid review of key concepts and articles in the area, to a comprehensive review of the topics.^20,21^ The Preferred Reporting Items for Systematic reviews and Meta-Analyse (PRISMA) extension for scoping reviews checklist (Appendix C) provides assurance that this scoping review details essential items pertinent to describing evaluations on physical activity programs.^22^ Using scoping review methodology to examine reviews of physical activity evaluations in North America since 2014, we provide a comprehensive description of evaluation frameworks, indicators, and measures to guide future program evaluations.

## Methods

### Data Sources

This review followed a protocol prepared by Goertzen and colleagues (2015)^23^  using methods similar to previous review articles completed by our research team.^24-27^ We conducted a systematic search of the following electronic databases: Medline, Scopus, Sportdiscus, Eric, PsycInfo, and CINAHL led by a health sciences librarian. The team developed a search strategy using controlled vocabulary and keywords to describe physical activity evaluations derived from WHO, Action Canada 20/20, and the DEDIPAC. Searches were performed in October 2018, with an updated systematic search occurring in July 2020. The search strategy is outlined in Appendix A Table A1

### Inclusion and Exclusion Criteria

**Figure 1.**
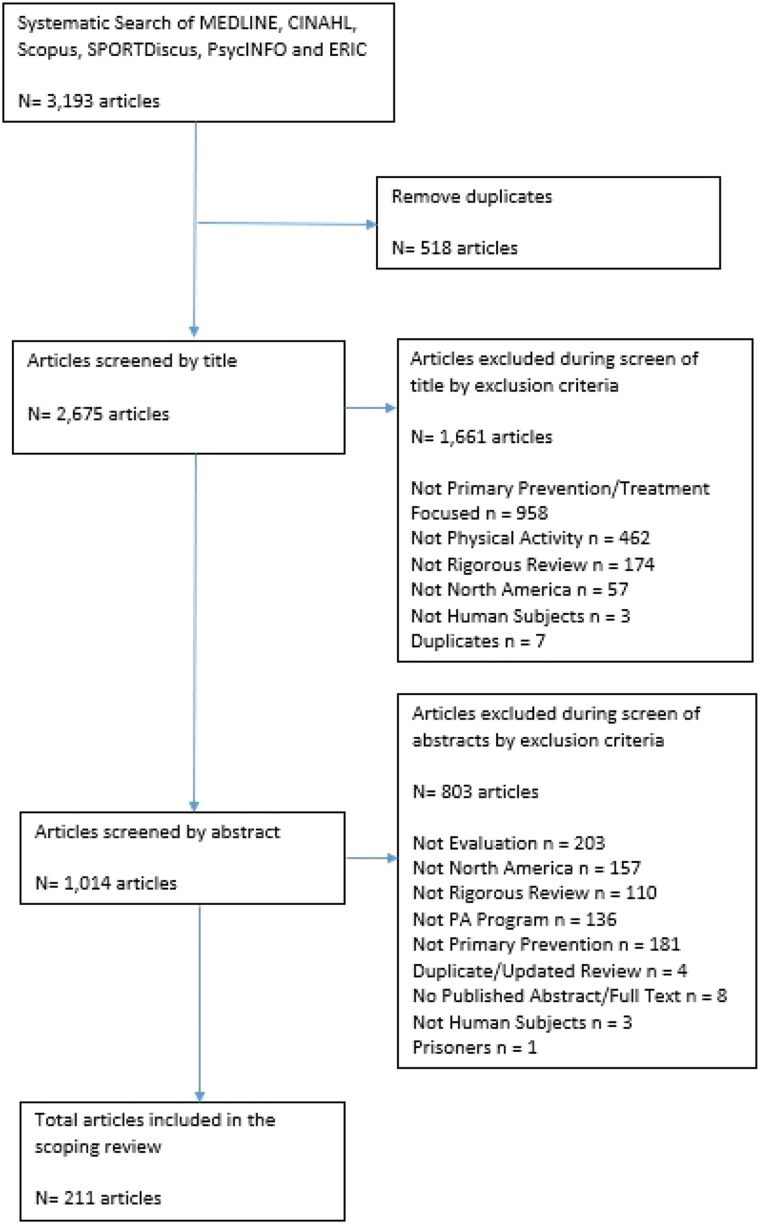
Population Targeted Within the Review by Evaluation Type.

We used a two-stage screening process. First, three reviewers screened the titles of all included reviews in Rayyan, an online application to assist with systematic reviews.^28^ Inclusion criteria included: review article, English language, human subjects, primary prevention focus, physical activity evaluation, and evaluations conducted in North America (which includes Canada, the United States of America, and Mexico) (Appendix A Table A1). Review articles considered for inclusion were systematic review, meta-analysis, meta-synthesis, scoping review, narrative review, rapid review, critical review, and integrative review. Any discrepancies were discussed, and when necessary resolved by a fourth reviewer. Following the screening of the article titles, articles included for abstract review were downloaded from Rayyan into an excel spreadsheet. Two researchers screened the abstracts of the remaining articles for inclusion based on the pre-determined criteria. If sufficient detail was not available in the abstract, the full text of the article was reviewed to determine eligibility for inclusion in the scoping review. Our systematic search yielded 3193 articles. After removing duplicate articles, we screened 2675 articles based on the inclusion/exclusion criteria. Following title and abstract screening there were 211 review articles (Appendix B Table A2) that met the inclusion criteria for the scoping review (PRISMA Flow Diagram provided in Figure 1) (Table A3).

### Data Extraction

Data was extracted from the abstract of each review article by two reviewers. Review articles described physical activity evaluations in North America. This description of the evaluation, as presented by the review article, was used to complete the data charting form. The data charting form was designed by the team and included the following:

(1) General detail (i.e., author, the type of review, number of studies included in the review, reported timeframe, location, and review objective/aim)
(2) Evaluation focus derived from the WHO Global Strategy on Diet, Physical Activity, and Health: A Framework to monitor and evaluate implementation^6,13^ and the Action Canada 20/20 Framework^14,15^:

- type of evaluation (implementation/process, output, short-term, intermediate, or long-term outcome),
- focus of the indicators: context (social inequity, disease burden, media, and built environment), settings (community, school, workplace, and media), and evidence

(3) Good practice characteristics for monitoring and evaluating a physical activity program as defined by DEDIPAC^17^ (i.e., costs considered (health benefit, behavior changes, intervention, policy, and economic), outcomes measured (physical, psychological, and both), effectiveness or efficiency sustainability, effect, reach, participant characteristics and generalizability, underlying processes, and active components)
(4) Evaluation framework, theory, and evaluation indicators. To sufficiently capture these areas the full text of each review article was assessed to complete the following columns in our data charting form: evaluation framework, theory, strategy, and what was measured. In addition to the theory name, when available reviewers documented if the theory guided the evaluation or program being evaluated.

### Data Synthesis

Two reviewers screened the titles and abstracts, extracted all data into the data extraction form, and reviewed and discussed all discrepancies to reach consensus. Descriptive statistics were used to summarize the categories within the data extraction form. Additionally, the two reviewers summarized narrative examples that could inform evaluation strategies. All authors reviewed and discussed preliminary results to reach consensus on the key findings.

## Results

There were 211 review articles published between January 2014 and July 2020 that collectively reviewed 8138 physical activity program evaluations. On average, there were 32 physical activity evaluation review articles each year. There were 5 physical activity evaluation review articles that focused exclusively on Canada,^29-33^ 90 articles that focused exclusively on the USA, and 116 articles that included studies from both Canada and the USA.

### Consideration When Conceptualizing and Developing an Evaluation Plan

#### Scientific Evidence

There were a number of different evaluation frameworks and theoretical approaches discussed within the included reviews. Stanhope et al^34^ point out that there are a variety of strong evaluation methods and measures; the decision on the approach to use should be tailored to the population and setting, and be based on the strengths and limitations of each approach. However, in the literature the terms “evaluation framework,” “theory,” and “strategy” were defined differently making comparisons between approaches difficult. For example, social cognitive theory was defined as a framework, a theoretical approach and a strategy among the included reviews (e.g., Refs. 35-37) For the purpose of this scoping review, we followed the US Department of Health and Human Services (HHS) definitions: (1) an evaluation framework is a guide to summarize and organize essential elements of program evaluation; (2) a theory is a set of beliefs used to understand change; and (3) a strategy is a method used to gather evidence.^12^

#### Evaluation Frameworks

Very few evaluations of physical activity programs described the use of a formal evaluation framework. This is not surprising as the majority of reviews were of randomized controlled trials that rarely include evaluation frameworks. There were a small number of frameworks used by physical activity evaluations, with Re-AIM being the most commonly referenced framework.^38-51^ Reviews reported that the RE-AIM framework was used to promote consistent reporting of intervention results from health promotion and disease management interventions by addressing multiple dimensions (populations, settings, and health conditions) and informing internal and external validity. Almost all of the articles in our review reported that evaluations focused on internal factors such as program effectiveness (95.7%), with external factors such as cost-effectiveness of the program infrequently considered (13.7%).

#### Theory

Fifty-nine of 211 (28%) reviews reported that evaluations included in their study referred to a specific theory that either informed the program evaluation or the program being evaluated. For example, authors mentioned that the socio-ecological model was used to assess the presence of each system in evaluation measures^52^ or was used to design the program to attend to each of the socio-ecological systems defined in the model.^53^ There were 26 different theoretical approaches mentioned by the reviews of physical activity evaluations; the most common were social cognitive theory, transtheoretical model/stages of change, socio-ecological model, theory of planned behavior, and the health belief model (Table 1).

**Table 1.** Theoretical Approaches Mentioned by Reviews.

Theory	Reference
Social cognitive theory	^35-37,43,46-48,52-78^
Transtheoretical model/stages of change	^35-37,47,48,51,52,55,56,61,62,66,68,72,75,79,80^
Socio-ecological model	^47,51-53,57-59,66,67,70,71,77,80-84^
Theory of planned behavior	^37,47,48,51,54,59,66,67,69,70,85^
Health belief model	^68-70,72,76,77,86^

**Figure 2.**
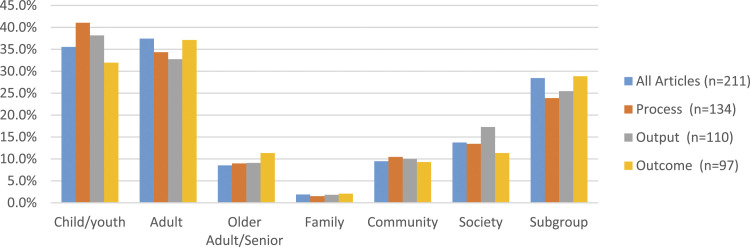
Population targeted within the review by evaluation type.

Universal and targeted approaches: populations and equity. Physical activity program evaluation reviews were primarily focused on individual adults (37.4%) and children and youth (35.5%). Population subgroups defined by ethnicity, disability, gender, and low-income were identified in 28.4% of reviews (Figure 2). Without capturing characteristics of the program participants, the significance of an evaluation’s findings to different groups cannot be determined.

### Evaluation Indicators

Evaluation indicators are specific, observable, and measurement statements to measure the program process and/or outcome.^12^

#### Process Indicators

Program delivery models each have their own unique strengths and limitations that must be considered for program implementation and evaluation.^54^ Process indicators describe and evaluate *how* an intervention was delivered.^12,13^ Process evaluations typically used the following measurement techniques to assess the program:

- Physical education observation (e.g., duration of strength training components, minutes of physical activity in the lesson, and interactions between the participants and instructor)
- Self-reported program adherence by participants (e.g., completion of home exercises)
- Program schedules (e.g., amount and type of physical activity)
- Program records (e.g., participation rate and retention rate)
- Program descriptors (length, priority population, setting, and type)

Process indicators were used in the majority (63.5%) of the included reviews. Process indicators included measures to assess program delivery, resource utilization, and external influences. For example, 38.8% of reviews reported that process indicators were able to identify aspects of a program model or program context (e.g., location and time, clinician proficiency, parental involvement, and peer involvement) that could inform future approaches to increase physical activity participation. Process indicators were used to assess fidelity of program implementation (66.4%) and the dose-response relationship (e.g., minutes of physical activity delivered vs received) was mentioned in 18.7% of reviews. Indicators of program reach were focused on uptake and adoption (32.0%), retention and adherence (30.6%), and access and engagement (20.9%).

**Figure 3.**
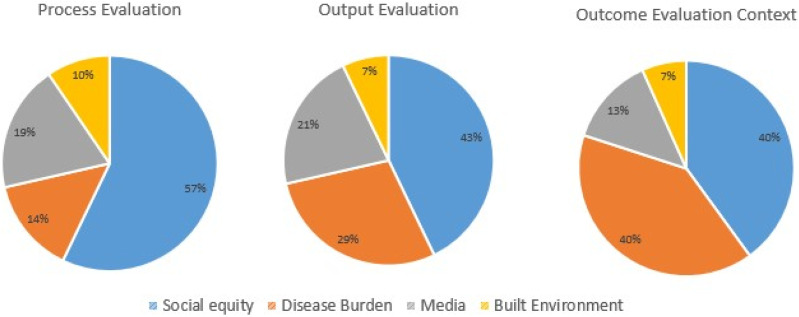
Issues related to national circumstances definded by the WHO by evaluation type.

When considering the focus of the process indicators,^13,14^ the majority (60.0%) of indicators in their review considered social inequity, while media-focused strategies (20.0%), disease burden (12.0%), or built environment (8.0%) were less prominent (Figure 3). Among articles that considered social inequity, ethnicity was the most common factor considered. Acknowledging the role of ethnicity in program development and implementation can influence program uptake as well as the ability of the program to retain participants and increase physical activity levels.^5,87^

Child/youth populations were the focus of 41.0% (55/134) of reviews of evaluations with process indicators; 26.9% (36/134) aimed to develop school-based physical activity programs and policies (Figure 2). For example, McKenzie and Smith^88^ describe the use of a System for Observing Fitness Instruction Time (SOFIT) in diverse settings to develop measures for assessing variations in programs developed for physical education. The measures include program structure/setting, teacher behaviors, and student characteristics.^88^

The built environment was mentioned by both the WHO and Active Canada 20/20; however, it was infrequently assessed in evaluations. Review articles that did assess the built environment found that commonly reported instruments used for measurement included geographical information systems (GIS), global positioning system (GPS), and neighborhood assessment.^89-91^ These instruments were used to assess characteristics of the built environment such as green space, accessibility of buildings, and walkability of the neighborhood. For example, McGrath et al reviewed evaluations that used GIS, GPS, and neighborhood assessments to measures the number of minutes of physical activity compared to number of meters to the closest neighborhood park and housing density per square kilometer.^89^

#### Outcome Indicators

Outcome indicators measure effects or changes resulting from the program and can be grouped into output (e.g., direct product of the activity), short-term outcomes (e.g., increased knowledge), intermediate outcomes (e.g., behavior change), or long-term outcomes (e.g., disease prevention and management).^12,13^

Output indicators focus on the immediate effect or product that results from the intervention.^11,12^ A majority (56.7%, 76/134) of the reviews that assessed evaluations with process indicators also included evaluations with output indicators. For example, one review describes the relationship between implementation processes (e.g., after-school, summer, and multiple times a day) and program outputs (including minutes of physical activity, fruit and vegetable consumption, and caloric intake) as found within 28 different program evaluations.^40^ Among reviews of evaluations with output indicators, 21.4% measured outputs such as self-reported increases in physical activity, number of people reached, number of sessions attended, and knowledge and attitudes towards behavior change (Figure 3). Some reviews also considered structures such as the built environment, and its influence on access to, and benefits gained from, physical activity programs (42.9%). For example, Calder et al^92^ explored evaluations that measured access of public indoor fitness centers by people with disabilities. This included researcher observations of the setting such as physical requirements needed to access equipment and bathrooms, as well as program availability, policies, and professionalism of staff.^92^ Societal level application of lessons learned from physical activity programs was the focus of 17.3% of reviews of evaluations with output indicators (Figure 2). For example, Hunter et al^93^ suggest the need for more urban green space areas following their review of physical activity program evaluations that demonstrated an increase in physical activity if the program encouraged physical activity (PA) in urban green space.^93^

Short-term, intermediate, and long-term outcome indicators measure the consequences from participating in the program and are typically measured months or years after the program. Disease burden was a large focus among these reviews (40.0%), largely aimed at determining long-term solutions for prevention of health conditions (Figure 3).

Almost half of the reviews in this review (46.0%, 97/211) included evaluations with outcome indicators. Intermediate outcomes were more likely to be assessed by review articles (70.1%, 68/97) compared to short-term (21.6%, 21/97) and long-term (35.1%, 34/97) outcomes. Evaluation guides, such as best practice guidelines for accelerometer use, have been developed to guide assessment of physical activity outcome measures.^94^ These guides aim to standardize how the instrument is used (e.g., location and time the device is worn) as well as the number of steps that represent being physically active.

Outcome measures can be categorized into the following groups: anthropometric, behavioral, or psychological (Table 2). Most evaluations measured physical outcomes (i.e., anthropometric and behavioral changes) following participation in a physical activity program (86.6%), with fewer evaluations assessing psychological or cognitive outcomes (36.1%). Arbour-Nicitopoulos et al^85^ reported that evaluations they reviewed used both physical (i.e., physical skill development) and psychological (i.e., psychological wellbeing) measures of outcomes for children and youth who participated in out-of-school physical activity programs. Psychological measures were more evident in evaluations focused on children and youth, older adults, or ethnic subpopulations. Some reviews also reported that evaluations used self-report (i.e., survey) measures for social support and relationships such as the CAR-DIA-2, 11 items or the Perceived Social Support Scale.^55^ Many of the reviews discussed evaluations with outcome indicators focused on determining if a physical activity program or strategy that was previously found to be effective would be effective in different population subgroups related to gender, disability, health conditions, or ethnicity/culture (40.0%).^55,87,95,96^ Table 2 provides a more detailed outline of the kinds of measures used in each categorical group. Finally, an extremely small proportion of reviews considered negative consequences of program participation (9.3%) despite evidence that negative consequences, including adverse events, physical injuries and falls, and worsening subjective wellbeing are important for designing programs and initiatives.^17^

**Table 2.** Outcome Indicators used within North American PA Program Evaluations.

Indicator Category	How It Is Measured	What Is Measured (Examples)
Anthropometric	Direct measure	• BMI, BMI percentile, and BMI z score
		• Waist circumference
		• % body fat
		• Skin folds
		• Blood pressure
		• Laboratory values (e.g., total cholesterol and blood glucose)
	Self-reported survey	• Weight and height
		• Weight change or weight loss
Behavioral	Monitor	• Accelerometer
		• Activity monitor
		• Pedometer
		• Step count
	Self-reported	• Summary score of physical activity
		• Subjective measures of physical activity (questionnaire or survey)
		o CHAMPS Physical Activity Questionnaire
		o 7-Day Activity Recall (7 Day PAR)
		o Godin Leisure Time Exercise Questionnaire
		o Paffenbarger activity questionnaire
		o Participant activity log
		o Physical Activity and Disability Scale (PADS)
		o International physical activity questionnaire
	Observation	• Presence of physical activity during the program (amount, length)
		• Exercise adherence reported by staff
		• Log books and site reported adherence
		• American Heart Association (AHA) indicators of cardiovascular wellbeing
Psychological	Self-reported	• Questionnaires and surveys developed by the evaluator for the specific program
		• Rosenberg Self-Esteem Scale
		• Depressive symptoms (Beck Depression Inventory)
		• Participant health Psychological Distress II and Psychological Well-Being II indices Perceived Stress Scale
		• Quality of well-being scale
		• Perceived general health and well-being
		• Self-efficacy for exercise behaviors, perceived barriers and perceived benefits
		• Readiness to Change
	Reported by parent or teacher	• Knowledge and attitudes, intrinsic motivation, competence, and autonomy
	Cognitive: Imaging	• MRI, fMRI, and electroencephalography
	Cognitive: Reported by teacher	• Learning outcomes of students reported by teachers

## Discussion

### Key Findings to Inform an Evaluation

This review of reviews found a wide variety of evaluation frameworks, theoretical underpinnings, program strategies, and evaluation measures used for different programs and settings. Variability, not only in the frameworks, theories, strategies, and measures, but also in how they were applied to each evaluation makes comparison between programs challenging, and may obscure emerging best practices. Consistency in defining the terms “evaluation framework,” “theory,” and “strategy” would support the use of evaluation findings in the development of new programs or improvement of current programs (Canadian Institute for Health Information).^12,35,96^ Authors should describe key aspects of their evaluation including use of frameworks, theories, strategies, and measures, define evaluation terms, and provide details of the program context to inform future evaluations and program development.^12^

High quality evaluations are commonly based on a specific framework that increases the likelihood the evaluation is developed appropriately and comprehensively to meet the specific identified needs.^12^ Three quarters of the reviews did not identify any specific evaluation frameworks. An evaluation framework promotes consistency in measurement (e.g., SOFIT) or reporting (e.g., RE-AIM) within program evaluations. In addition to utilizing an evaluation framework, incorporation of a theoretical approach can be important to ensure attention to factors that influence physical activity participation and may impact program effectiveness.^35^ Future research should assess and compare the application of evaluation frameworks and theories within the published evaluation literature to inform approaches for future evaluations.

The characteristics of the population of interest such as race, income, and geography should be considered as they impact program participation.^5,6,11-15^ Populations from lower socioeconomic groups are less physically active compared to those from higher socioeconomic groups.^5^ Although over half of the reviews included equity as an important consideration for primary prevention, only 30% of the articles in this scoping review considered participant characteristics and equitable distribution of physical activity strategies. Thus, understanding the characteristics of the at-risk population a program is designed to address is important, independent of the type of evaluation. In contrast to this, more than three-quarters of the reviews in this scoping review reported on the effect size of interventions; however, we would argue that contextual data is necessary to understand not only whether a particular program or intervention “works” but for whom it works and under what conditions. Equity considerations and targeted programming can address the challenges of participating in physical activity among populations least likely to be active or more likely to experience barriers to physical activity participation.^5,14^ The application of an equity lens including socioeconomic status and sociocultural aspects, such as gender, ethnicity, religion, culture, migrant status, neighborhood characteristics, and social capital, can inform population health interventions.^87^ Describing the population informs the usefulness, feasibility, fairness, and accuracy of an evaluation plan.^12^ Evaluation measures should include both specific measures of physical activity and sociodemographic, cultural, economic, political, and geographic factors that impact participation.^5,12^

### Strengths and Opportunities in the Evaluation Literature

#### Process Indicators

Reviews of evaluations with process indicators proposed future directions for physical activity programs (i.e., strategies and policies) and evaluation tools (i.e., frameworks and standardized instruments) to provide information that can enable the development of context-specific strategies. According to the *Introduction to Program Evaluation for Public Health Programs* new programs should use an implementation/process evaluation to assess program implementation and/or examine contextual factors that could affect program activities.^12^ The evaluation may include some output or short-term outcome indicators.^6,13^ Over half of the articles in this review included process indicators and many of these also included output indicators. Together these process indicators suggest the use of standardized methods and a combination of observation, self-reported measures and document review to evaluate programs and inform strategies that can be implemented at a societal level.

Active Canada 20/20^14,15^ and WHO^5^ emphasize the importance of the built environment on physical activity participation. However, the built environment and social infrastructure were infrequently considered within physical activity evaluations. Measuring the influence of the built environment can inform city planning, policy, and physical activity funding streams. Although some tools have been suggested for measurement of the built environment on PA participation, future research should continue to develop these tools.^90^

#### Outcome Indicators

An outcome evaluation can be conducted as soon as the desired outcome(s) can be expected to have occurred (e.g., accelerometer measurements at 6 months or longer can predict the intermediate and long-term outcomes of the program).^12,13,56,57^ However, similar to Ling et al, we found that many reviews conducted evaluations within 6 months of the intervention, and few evaluated long-term outcomes of their program.^57^ Several organizations posited that evaluations should consider both physical and psychological outcomes, and include possible positive and negative consequences of participating.^12,13,15,85^ However, reviews in this scoping review focused largely on physical outcomes (i.e., anthropometric measures and amount of physical activity) with very few considering psychological outcomes and negative consequences of participation. Similar to McGoey et al,^46^ almost all of the reviews of evaluations in our review focused solely on determining if a program effectively increased physical activity without considering cost-effectiveness, effect size, or generalizability.^46^

### Strengths and Limitations of This Review

This scoping review provides a comprehensive review of physical activity evaluation literature in North America. Informed by current guidelines (e.g., WHO, Active Canada 20/20, and HHS) our review systematically searched for peer-reviewed published review articles that summarized physical activity evaluations. Peer-reviewed literature has been assessed by experts in the field for quality and completeness.

This scoping review is a “review of reviews,” and each review included a number of physical activity evaluations. The reviews we included may not have fully detailed all key elements in the evaluation designs they examined, and so we were limited to what was reported in the reviews. Further examination of the original evaluation articles may provide additional details on the evaluation framework used to assess the physical activity program.

To obtain a larger breadth of data, the data extraction process focused on obtaining study details through abstract review. This may also have affected the depth of detail obtained from each study. However, the full article of each review was assessed for evaluation framework(s), theories, and measures to ensure there were minimal gaps in the presented information on these topics.

## Conclusions

Comprehensive evaluation designs support physical activity program improvement as well as the development and expansion of well-designed programs and strategies. This scoping review provides a comprehensive overview of how the frameworks, theories, strategies, indicators, and available measures and tools have been utilized in physical activity evaluation in North America. Based on the findings in this review, the creation of a plain language practice-based guide might contribute to greater use of and more robust physical activity evaluations. Capturing participant characteristics within evaluation literature would also help inform universal and targeted approaches for physical activity promotion. Future reviews should be sure to include precise descriptions of the guiding theory, frameworks, strategy, and indicators used in any specific program to add clarity and make the effectiveness of said program easier to quantify. Contextual factors, positive and negative outputs/outcomes, the use of evaluation frameworks, and measures of program sustainability can further inform future evaluations, which in turn provide an evidence-base for physical activity programming, policy, and funding.

### So What? Implications for Health Promotion Practitioners and Research

#### What is Already Known on This Topic?

Physical inactivity is pervasive and negatively impacts health. Multiple programs attempt to increase physical activity. Robust program evaluations can identify effective promotion strategies; however, it is unclear to what extent existing evaluation frameworks are being applied.

#### What Does This Article Add?

This scoping review of reviews systematically maps and describes evaluations of physical activity programs to summarize key characteristics of the published literature and suggest opportunities to strengthen current evaluations.

#### What Are the Implications for Health Promotion Practice and Research?

We describe review characteristics, evaluation measures and “good practice characteristics” to inform evaluation strategies. This review defines current terminology and describes the frameworks and measures that have been applied in physical activity evaluations. Contextual factors, negative outcomes, and measures of program sustainability would strengthen future evaluations and provide an evidence-base for physical activity programming, policy, and funding.

## Appendix A. Ovid MEDLINE(R) and Epub Ahead of Print, In-Process, and Other Non-Indexed Citations and Daily 1946 to July 2020

1. exp exercise/

2. exp Physical Fitness/

3. exercise*.kw. or exercise intervention*.tw,kw.

4. physical activit*.tw,kw.

5. physical fitness.tw,kw.

6. physically fit.tw,kw.

7. or/1-6 [PA string]

8. intervention*.tw,kw.

9. program*.tw,kw.

10. (policy or policies).tw,kw.

11. promot*.tw,kw.

12. approach*.tw,kw.

13. environment*.tw,kw.

14. strateg*.tw,kw.

15. practice*.tw,kw.

16. exp Public Policy/

17. exp Health Promotion/

18. Health Plan Implementation/

19. or/8-18 [intervention program policy string]

20. Program Evaluation/

21. "Outcome Assessment (Health Care)"/

22. (evaluat* adj3 implement*).tw,kw.

23. (evaluat* adj2 framework).tw,kw.

24. good practice*.tw,kw.

25. best practice*.tw,kw.

26. recommended practice*.tw,kw.

27. recommended strateg*.tw,kw.

28. recommendation* for practice*.tw,kw.

29. or/20-28 [evaluation string]

30. indicator*.tw,kw.

31. factor*.tw,kw.

32. barrier*.tw,kw.

33. facilitat*.tw,kw.

34. framework*.tw,kw.

35. or/30-34 [indicator string]

36. exp North America/

37. north america*.tw,kw.

38. (canada or canadian*).tw,kw,cp.

39. united states.tw,kw,cp.

40. america*.tw,kw.

41. (mexican or mexico).tw,kw,cp.

42. or/36-41 [north american string]

43. scoping review*.tw,kw.

44. scoping stud*.tw,kw.

45. rapid review*.tw,kw.

46. systematic review*.tw,kw.

47. meta-analysis.tw,kw.

48. meta-synthesis.tw,kw.

49. critical review.tw,kw.

50. (qualitative adj2 review).tw,kw.

51. mapping review.tw,kw.

52. mixed methods review.tw,kw.

53. umbrella review.tw,kw.

54. or/43-53 [review types string]

55. 7 and 19 and (29 or 35) and 42

56. 55 and 54

57. limit 55 to (meta analysis or systematic reviews)

58. 56 or 57

59. 58 not (exp animals/not humans/)

60. limit 59 to (english language and yr="2014 -Current")

### Appendix B

#### Appendix C

**Table A1.** Inclusion and exclusion criteria for the scoping review

Inclusion Criteria	Exclusion Criteria
• Published in English between January 2014 and July 2020	• Journal articles that are not rigorous reviews (i.e., outside of those defined in the inclusion list), such as book reviews, opinion articles, commentaries, or editorial reviews
• Human subjects of all age groups	
• Primary prevention: Research that targets the general population and only randomly includes individual with illness, disease, or conditions	• Targeting treatment of a specific disease, illness or condition
• Review articles: Systematic review, meta-analysis, meta-synthesis, scoping review, narrative review, rapid review, critical review, and integrative review	
• Focused on physical activity program evaluation	• Reviews with less than 50% of the articles located in North America
• Research in North America	

**Table A2.** Review Articles included in this Scoping Review

Reference
Albarracín, D., Wilson, K., Chan, MP., Durantini, M., & Sanchez, F. (2018). Action and inaction in multi-behavior recommendations: A meta-analysis of lifestyle interventions. *Health Psychol Rev, 12*(1), 1-24. doi: 10.1080/17437199.2017.1369140
Alharbi, M., Straiton, N., Smith, S., Neubeck, L., & Gallagher, R. (2019). Data management and wearables in older adults: A systematic review. *Maturitas, 124*, 100-110. doi: 10.1016/j.maturitas.2019.03.012
Allen, JK., Stephens, J., & Patel, A. (2014). Technology-assisted weight management interventions: Systematic review of clinical trials. *Telemed J E Health, 20*(12), 1103-1120. doi: 10.1089/tmj.2014.0030
Allen, R., Rogozinska, E., Sivarajasingam, P., Khan, KS., &Thangaratinam, S. (2014). Effect of diet- and lifestyle-based metabolic risk-modifying interventions on preeclampsia: a meta-analysis. *Acta obstetricia et gynecologica Scandinavica, 93*(10), 973-985. doi: 10.1111/aogs.12467
Állison. RL. (2017). Back to basics: The effect of healthy diet and exercise on Chronic disease management. South Dakota Medicine, 10-18
Alvarez-Bueno, C., Pesce, C., Cavero-Redondo, I., Sanchez-López, M., Martínez-Hortelano, JA., & Martínez-Vizcaíno, V. (2017). The effect of physical activity interventions on Children’s cognition and metacognition: A systematic review and meta-analysis. *Journal of the American Academy of Child and Adolescent Psychiatry, 56*(9), 729-738. doi: 10.1016/j.jaac.2017.06.012
Aneni, EC., Roberson, LL., Maziak, W., Agatston, AS., Feldman, T., Rouseff, M., … Nasir, K. (2014). A systematic review of internet-based worksite wellness approaches for cardiovascular disease risk management: Outcomes, changes and opportunities. PLoS One, 9(3):e92759. https://doi.org/10.1371/journal.pone.0092759
Appelhans, BM., Moss, OA., & Cerwinske, LA. (2016). Systematic review of paediatric weight management interventions delivered in the home setting. *Obesity Reviews, 17*(10), 977-988. doi: 10.1111/obr.12427
Arbour-Nicitopoulos, KP., Grassmann, V., Orr, K., McPherson, AC., Faulkner, GE., & Wright, FV. (2018). A scoping review of inclusive out-of-school time physical activity programs for children and youth with physical disabilities. *Adapted Physical Activity Quarterly, 35*(1):111-138. doi: 10.1123/apaq.2017-0012
Ashdown-Franks, G., Williams, J., Vancampfort, D., Firth, J., Schuch, F., Hubbard, K., . . . Stubbs, B. (2018). Is it possible for people with severe mental illness to sit less and move more? A systematic review of interventions to increase physical activity or reduce sedentary behavior. *Schizophrenia Research, 202*, 3-16. doi: 10.1016/j.schres.2018.06.058
Ashton, LM., Morgon, PJ., Hutchesson, MJ., Rollo, ME., Young, MD., & Collins, CE. (2015). A systematic review of SNAPO (smoking, nutrition, alcohol, physical activity and obesity) randomized controlled trials in young adult men. *Preventive Medicine, 81*, 221-231. doi: 10.1016/j.ypmed.2015.09.005
Assumpção Picorelli, AM. Máximo Pereira, LS., Sirineu Pereira, D., Felício, D., & Sherrington, C. (2014). Adherence to exercise programs for older people is influenced by program characteristics and personal factors: A systematic review. *Journal of Physiotherapy, 60*(3), 151-156. doi: 10.1016/j.jphys.2014.06.012
Bailie, L., Loughrey, MB., & Coleman, HG. (2017). Lifestyle risk factors for serrated colorectal polyps: A systematic review and meta-analysis. *Gastroenterology, 152*(1), 92-104. doi: 10.1053/j.gastro.2016.09.003
Baillie, CPT., Galaviz, KI., Emiry, K., Bruner, MW., Bruner, BG., & Lévesque, L. (2017). Physical activity interventions to promote positive youth development among indigenous youth: A RE-AIM review. Transl Behav Med, 7(1), 43-51. doi: 10.1007/s13142-016-0428-2
Baker, A., Sirois-Leclerc, H., & Tolloch, H. (2016). The impact of long-term physical activity interventions for overweight/obese postmenopausal women on adiposity indicators, physical Capacity, and mental health Outcomes: A systematic review. *Journal of* Obesity, 2016(8), 1-22. doi: 10.1155/2016/6169890
Barha, CK., Davis, JC., Falck, RS., Nagamatsu, LS., & Liu-Ambrose, T. (2017). Sex differences in exercise efficacy to improve cognition: A systematic review and meta-analysis of randomized controlled trials in older humans. *Frontiers in Neuroendocrinology, 46*, 71-85. doi: 10.1016/j.yfrne.2017.04.002
Barr-Anderson, DJ., Singleton, C., Cotwright, CJ., Floyd, MF., & Affuso, O. (2014). Outside-of-school time obesity prevention and treatment interventions in African American youth. Obesity Reviews, 15(Supp4), 26-45. doi: 10.1111/obr.12204
Bassett-Gunter, R., Angevaare, K., Tomasone, J., Leo, J., Varughese, B., Langvee, J., … Martin Ginis, K. (2019). A systematic scoping review: Resources targeting the training and education of health and recreation practitioners to support physical activity among people with physical disabilities. *Disability and Health Journal, 12*(4), 542-550. https://dx.doi.org/10.1016/j.dhjo.2019.06.007
Beall, RF., Baskerville, N., Golfam, M., Saeed, S., & Little, J. (2014). Modes of delivery in preventive intervention studies: A rapid review. *European Journal of Clinical Investigation, 44*(7), 688-696. https://doi.org/10.1111/eci.12279
Beasley, JM., Wagnild, JM., Pollard, TM., Roberts, TR., & Ahkter, N. (2020). Effectiveness of diet and physical activity interventions among Chinese-origin populations living in high income countries: A systematic review. *BMC Public Health, 20*(1),1019-1044. doi: 10.1186/s12889-020-08805-3
Beauchamp, MK., Lee, A., Ward, RF., Harrison, SM., Bain, PA., Goldstein, RS., … Jette, AM. (2017). Do exercise interventions improve participation in life roles in older adults? A systematic review and meta- analysis. *Physical Therapy, 97*(10), 964-974. doi: 10.1093/ptj/pzx082
Beisbier, S., & Laverdure, P. (2020). Occupation- and activity-based interventions to improve performance of instrumental activities of daily living and Rest and Sleep for children and youth ages 5-21: A systematic review. The American Journal of Occupational Therapy, 74(2), 1-32. doi: 10.5014/ajot.2020.039636
Beishuizen, CRL., Stephan, BCM., van Gool, WA., Brayne, C., Peters, RJG., Andrieu, S., … Richard, E. (2016). Web-based interventions targeting cardiovascular risk factors in middle-aged and older people: A systematic review and meta-analysis. *Journal of Medical Internet Research*, 18(3), e55. doi:10.2196/jmir.5218
Bender, MS., Choi, J., Won, GY., & Fukuoka, Y. (2014). Randomized controlled trial lifestyle interventions for Asian Americans: A systematic review. *Preventive Medicine, 67*, 171-181. doi: 10.1016/j.ypmed.2014.07.034
Berger, AA., Peragallo-Urrutia, R., & Nicholson, WK. (2014). Systematic review of the effect of individual and combined nutrition and exercise interventions on weight, adiposity and metabolic outcomes after delivery: Evidence for developing behavioral guidelines for post-partum weight control. *BMC Pregnancy and Childbirth, 14*(1), 319. doi: 10.1186/1471-2393-14-319
Bergeron, CD., Tanner, AH., Friedman, DB., Zheng, Y., Schrock, CS., Bornstein, DB., … Swift, N. (2019). Physical activity communication: A scoping review of the literature. *Health Promotion Practice, 20*(3), 344-353. doi: 10.1177/1524839919834272
Bhuiyan, N., Singh, P., Harden, SM., & Mama, SK. (2019). Rural physical activity interventions in the United States: a systematic review and RE-AIM evaluation. *International Journal of Behavioral Nutrition and Physical Activity*, 16(1), 140. https://doi.org/10.1186/s12966-019-0903-5
Bian, RR., Piatt, GA., Sen, A., Plegue, MA., De Michele, ML., Hafez, D., … Richardson, CR. (2017). The effect of technology-mediated diabetes prevention interventions on weight: A meta-analysis. *Journal of Medical Internet Research, 19*(3):e76. doi: 10.2196/jmir.4709
Biddle, SJH., Braithwaite, R., & Pearson, N. (2014). The effectiveness of interventions to increase physical activity among adolescent girls: A meta-analysis. *Preventive Medicine, 62*, 119-131. doi: 10.1016/j.ypmed.2014.02.009
Blaga, OM., Vasilescu, L., & Chereches, RM. (2018). Use and effectiveness of behavioural economics in interventions for lifestyle risk factors of non-communicable diseases: a systematic review with policy implications. *Perspectives in Public Health, 138*(2), 100-110. doi: 10.1177/1757913917720233
Bock, C., Jarczok, MN., & Litaker, D. (2014). Community-based efforts to promote physical activity: A systematic review of interventions considering mode of delivery, study quality and population subgroups. *Journal of Science and Medicine in Sport, 17*(3), 276-282. doi: 10.1016/j.jsams.2013.04.009
Bragg, E., & Pritchard, WL. (2019). Wheelchair physical activities and sports for children and adolescents: A scoping review. *Physical and Occupational Therapy in Pediatrics, 39*(6), 567-579. doi: 10.1080/01942638.2019.1609151
Brickwood, KJ., Watson, G., O-Brien, J., & Willaims, AD. (2019). Consumer-based wearable activity trackers increase physical activity participation: Systematic review and meta-analysis. *JMIR mHealth and uHealth, 7*(4), e11819. https://dx.doi.org/10.2196/11819
Brown, SA., García, AA., Zuñiga, JA., Lewis, KA. (2018). Effectiveness of workplace diabetes prevention programs: A systematic review of the evidence. *Patient Education and Counseling, 10*(6), 1036-1050. https://doi.org/10.1016/j.pec.2018.01.001
Brown, T., Moore, T. H., Hooper, L., Gao, Y., Zayegh, A., Ijaz, S., . . . Summerbell, C. D. (2019). Interventions for preventing obesity in children. *Cochrane Database of Systematic Reviews, 7*(7), CD001871. doi: 10.1002/14651858.CD001871.pub4
Buchanan, LR., Rooks-Peck, CR., Finnie, RKC., Wethington, HR., Jacob, V., Fulton, JE., … Glanz, K. (2016). Reducing recreational sedentary screen time: A community guide systematic review. *American Journal of Preventive Medicine, 50*(3), 402-415. doi: 10.1016/j.amepre.2015.09.030
Bull, ER., McCleary, N., Li, X., Dombrowski, SU., Dusseldorp, E., & Johnston, M. (2018). Interventions to promote healthy eating, physical activity and smoking in low-income groups: a systematic review with meta-analysis of behavior change techniques and delivery/context. *International Journal of Behavioral Medicine, 25*(6), 605-616. doi: 10.1007/s12529-018-9734-z
Bull, ER., Dombrowski, SU., McCleary, N, & Johnston, M. (2014). Are interventions for low-income groups effective in changing healthy eating, physical activity and smoking behaviours? A systematic review and meta-analysis. *BMJ Open, 4*(11), e006046. doi: 10.1136/bmjopen-2014-006046
Burns, RD., Fu, Y., & Zhang, P. (2019). Resistance training and insulin sensitivity in youth: A meta-analysis. *American Journal of Health Behavior, 43*(2), 228-242. https://dx.doi.org/10.5993/AJHB.43.2.1
Buttazzoni, AN., Van Kesteren, ES., Shah, TI., & Gilliand, JA. (2018). Active school travel intervention methodologies in north America: A systematic review. American Journal of *Preventive Medicine, 55*(1), 115-124. doi: 10.1016/j.amepre.2018.04.007
Calder, A., Sole, G., & Mulligan, H. (2018). The accessibility of fitness centers for people with disabilities: A systematic review. *Disability Health Journal, 11*(4), 525-536. https://doi.org/10.1016/j.dhjo.2018.04.002
Campbell, WW., Kraus, WE., Powell, KE., Haskell, WL., Janz, KF., Jakicic, JM., . . . Bartlett, DB. (2019). High-intensity interval training for cardiometabolic disease prevention. *Medicine and Science in Sports and Exercise, 51*(6), 1220-1226. 10.1249/MSS.0000000000001934
Carter, DD., Robinson, K., Forbes, J., & Hayes, S. (2018). Experiences of mobile health in promoting physical activity: A qualitative systematic review and meta-ethnography. *PloS One, 13*(12), e0208759. https://dx.doi.org/10.1371/journal.pone.0208759
Casey, B., Coote, S., Shirazipour, C., Hannigan, A., Motl, R., Martin, G., … Latimer-Cheung, A. (2017). Modifiable psychosocial constructs associated with physical activity participation in people with multiple sclerosis: A systematic review and meta-analysis. *Archives in Physical Medicine and Rehabilitation, 98*(7), 1453-1475. doi: 10.1016/j.apmr.2017.01.027
Cassar, S., Salmon, J., Timperio, A., Naylor, PJ., Van Nassau, F., Contardo Ayala, AM., … Koorts H. (2019). Adoption, implementation and sustainability of school-based physical activity and sedentary behavior interventions in real-world settings: A systematic review. *International Journal of Behavioral Nutrition and Physical Activity*, 16(1), 120. https://doi.org/10.1186/s12966-019-0876-4
Castro, O., Ng, K., Novoradovskay, E., Bosselut, G., & Hassandra, M. (2018). A scoping review on interventions to promote physical activity among adults with disabilities. *Disabil Health J, 11*(2), 174-183. doi: 10.1016/j.dhjo.2017.10.013
Champion, KE., Parmenter, B., McGowan, C., Spring, B., Wafford, Q. E., Gardner, L. A., . . . The Health4Life, t. Effectiveness of school-based eHealth interventions to prevent multiple lifestyle risk behaviours among adolescents: a systematic review and meta-analysis. The Lancet Digital health, 2-19;1(5), e206-e221. 10.1016/S2589-7500(19)30088-3
Clark, JE. (2015). Diet, exercise or diet with exercise: Comparing the effectiveness of treatment options for weight-loss and changes in fitness for adults (18–65 years old) who are overfat, or obese; systematic review and meta-analysis. *Journal of Diabetes and Metabolic Disorders, 14*(1), 31. doi: 10.1186/s40200-015-0154-1
Cooper, C., Gross, A., Brinkman, C., Pope, R., Allen, K., Hastings, S. … Goode, AP. (2018). The impact of wearable motion sensing technology on physical activity in older adults. *Experimental Gerontology, 112*, 9-19. doi: 10.1016/j.exger.2018.08.002
Corona, E., Flores, YN., & Arab, L. (2016). Trends in evidence-based lifestyle interventions directed at obese and overweight adult latinos in the US: A systematic review of the literature. *Journal of Community Health, 41*(3), 667-673. doi: 10.1007/s10900-015-0119-9
Cotie, LM., Prince, SA., Elliott, CG., Ziss, MC., McDonnell, LA., Mullen, KA., … Reed, JL. (2018). The effectiveness of eHealth interventions on physical activity and measures of obesity among working-age women: a systematic review and meta-analysis. *Obesity Reviews, 19*(10), 1340-1358. doi: 10.1111/obr.12700
Cradock, AL., Barrett, JL., Kenney, EL., Giles, CM., Ward, ZJ., Long, MW., … Gortmaker, SL. (2017). Using cost-effectiveness analysis to prioritize policy and programmatic approaches to physical activity promotion and obesity prevention in childhood. *Preventive Medicine, 95*, S17-S27. doi: 10.1016/j.ypmed.2016.10.017
Cuthbert, CA., King-Shier, K., Ruether, D., Tapp, DM., & Culos-Reed, SN. (2017). What is the state of the science on physical activity interventions for family caregivers? A systematic review and RE-AIM evaluation. *J Phys Act Health*. 14(7) 578-595. doi: 10.1123/jpah.2016-0280
Dabravolskaj, J., Montemurro, G., Ekwaru, JP., Wu, XY., Storey, K., Campbell, S., … Ohinmaa, A. (2020). Effectiveness of school-based health promotion interventions prioritized by stakeholders from health and education sectors: A systematic review and meta-analysis. *Preventive Medicine Reports*, 19, 1-18. https://doi.org/10.1016/j.pmedr.2020.101138
Daly-Smith, AJ., Zwolinsky, S., McKenna, J., Tomporowski, PD., Defeyter, MA., & Manley, A. (2018). Systematic review of acute physically active learning and classroom movement breaks on children’s physical activity, cognition, academic performance and classroom behavior: Understanding critical design features. *BMJ Open Sport and Exercise Medicine, 4*(1), e000341. doi: 10.1136/bmjsem-2018-000341
de Roon, M., May, AM., McTiernan, A., Scholten, RJPM., Peeters, PHM., Friedenreich, CM., … Monninkhof, EM. (2018). Effect of exercise and/or reduced calorie dietary interventions on breast cancer-related endogenous sex hormones in healthy postmenopausal women. Breast cancer research, 20(1), 81-97. doi: 10.1186/s13058-018-1009-8
Delva, S., Waligora Mendez, KJ., Cajita, M., Koirala, B., Shan, R., Wongvibulsin, S., . . . Han, H-R. (2020). Efficacy of mobile health for self-management of cardiometabolic risk factors: A theory-guided systematic review. *Journal of Cardiovascular Nursing,* 2020. doi: 10.1097/JCN.0000000000000659
Dillon, SR., Adams, D., Goudy, L., Bittner, M., & McNamara, S. (2016). Evaluating exercise as evidence-based practice for individuals with autism spectrum disorder. *Frontiers in Public Health*, 4, 290-299. doi: 10.3389/fpubh.2016.00290
Dinoff, A., Herrmann, N., Swardfager, W., & Lanctôt, K. (2017). The effect of acute exercise on blood concentrations of brain-derived neurotrophic factor in healthy adults: a meta-analysis. European Journal of Neuroscience, 46(1), 1635-1646. doi: 10.1111/ejn.13603
Edwards, N., & Dulai, J. (2018). Examining the relationships between walkability and physical activity among older persons: What about stairs? *BMC Public Health, 18*(1), 1025. https://doi.org/10.1186/s12889-018-5945-0
Ekelund, U., Tarp, J., Steene-Johannessen, J., Hansen, BH., Jefferis, B., Fagerland, MW., . . . Lee, IM. (2019). Dose-response associations between accelerometry measured physical activity and sedentary time and all cause mortality: Systematic review and harmonised meta-analysis. *The BMJ, 366*, 14570. doi: 10.1136/bmj.l4570
Errisuriz, VL., Golaszewski, NM., Born, K., & Bartholomew, JB. (2018), systematic review of physical education-based physical activity interventions among elementary school children. *Journal of Primary Prevention, 39*(2), 303-327. doi: 10.1007/s10935-018-0507-x
Farren, L., Belza, B., Allen, P., Brolliar, S., Brown, D., Cormier, ML., … Rosenberg, DE. (2015). Mall walking program environments, features and participants: A scoping review. *Preventing Chronic Disease* 12, e129. doi: http://dx.doi.org/10.5888/pcd12.150027
Finlay, A., Wittert, G., & short, CE. (2018). A systematic review of physical activity-based behavior change interventions reaching men with prostate cancer. *Journal of Cancer Survival, 12*(4), 571-591. doi: 10.1007/s11764-018-0694-8
Fox, J., Rioux, BV., Goulet, EDB., Johanssen, NM., Swift, DL., Bouchard, DR., … Sénéchal, M. (2018). Effect of an acute exercise bout on immediate post-exercise irisin concentration in adults: A meta-analysis. Scandinavian Journal of Medicine and science in Sports, 28(1), 16-28. https://doi.org/10.1111/sms.12904
Frerichs, L., Ataga, O., Corbie-Smith, G., & Lindau, ST. (2016). Child and youth participatory interventions for addressing lifestyle-related childhood obesity: a systematic review. *Obesity Reviews, 17*(12), 1276-1286. doi: 10.1111/obr.12468
Frey, GC., Temple, VA., & Stanish, HI. (2017). Interventions to promote physical activity for youth with intellectual disabilities. Salud Publica de Mexico, 59(4), 437-445. doi: 10.21149/8203
Funderburk, JS., Shepardson, RL., Wray, J., Acker, J., Beehler, GP., Possemato, K., … Maisto, SA. (2018). Behavioral medicine interventions for adult primary care settings: A review. *Families, Systems and Health, 36*(3), 368-399. doi: 10.1037/fsh0000333
Gao, Z., Chen, S., Pasco, D., & Pope, Z. (2015). A meta-analysis of active video games on health outcomes among children and adolescents. *Obesity Reviews, 16*(9), 783-794. doi: 10.1111/obr.12287
Gayes, LA., & Steele, RG. (2014). A meta-analysis of motivational interviewing interventions for pediatric health behavior change. *Journal of Consulting and Clinical Psychology, 82*(3), 521-535. doi: 10.1037/a0035917
Goode, AP., Hall, KS., Batch, BC., Huffman, KM., Hastings, SN., Allen, KD., … Gierisch, JM. (2017). The impact of interventions that integrate accelerometers on physical activity and weight loss: A systematic review. *Annals of Behavioral Medicine, 51*(1), 79-93. https://doi.org/10.1007/s12160-016-9829-1
Green, AC., Hayman, LL., & Cooley, ME. (2015). Multiple health behavior change in adults with or at risk for cancer: A systematic review. American Journal of Health behavior, 39(3), 380-394. doi: 10.5993/AJHB.39.3.11
Hardee, JP., & Fetters, L. (2017). The effect of exercise intervention on daily life activities and social participation in individuals with down syndrome: A systematic review. *Research on Developmental Disabilities, 62*, 81-103. doi: 10.1016/j.ridd.2017.01.011
Healy, S., Nacario, A., Braithwaite, RE., & Hopper, C. (2018). The effect of physical activity interventions on youth with autism spectrum disorder: A meta-analysis. *Autism Research, 11*(6), 818-833. doi: 10.1002/aur.1955
Heerman, WJ., JaKa, MM., Berge, JM., Trapl, ES., Sommer, EC., Samuels, LR., … Barkin, SL. (2017). The dose of behavioral interventions to prevent and treat childhood obesity: A systematic review and meta-regression. *International Journal of Behavioral Nutrition and Physical Activity, 14*(1), 157. doi: 10.1186/s12966-017-0615-7
Higgins, TJ., Middleton, KR., Winner, L., & Janelle, CM. (2014). Physical activity interventions differentially affect exercise task and barrier self-efficacy: a meta-analysis. *Health Psychology, 33*(8), 891-903. doi: 10.1037/a0033864
Hinkley, T., Teychenne, M., Downing, KL., Ball, K., Salmon, J., & Hesketh, KD. (2014). Early childhood physical activity, sedentary behaviors and psychosocial well-being: a systematic review. *Preventive Medicine, 62*, 182-192. doi: 10.1016/j.ypmed.2014.02.007
Hirsch, JA., DeVries, DN., Brauer, M., Frank, LD., & Winters, M. (2018). Impact of new rapid transit on physical activity: A meta-analysis. *Preventive Medicine Reports, 10*, 184-190. https://doi.org/10.1016/j.pmedr.2018.03.008
Hunter, RF., Christian, H., Veitch, J., Astell-Burt, T., Hipp, JA., & Schipperijn, J. (2015). The impact of interventions to promote physical activity in urban green space: A systematic review and recommendations for future research. *Social Science and Medicine, 124*, 246-256. doi: 10.1016/j.socscimed.2014.11.051
Husk, K., Lovell, R., Cooper, C., Stahl-Timmins, W., & Garside, R. (2016). Participation in environmental enhancement and conservation activities for health and well-being in adults: A review of quantitative and qualitative evidence. *Cochrane database of systematic reviews, 2016*(5), CD01351. doi: 10.1002/14651858.CD010351.pub2
Jenkins, F., Jenkins, C., Gregoski, MJ., & Magwood, GS. (2017). Interventions promoting physical activity in African american women: An integrative review. *Journal of Cardiovascular Nursing, 32*(1), 22-29. doi: 10.1097/JCN.0000000000000298
Jirikowic, TL., & Kerfeld, CI. (2016). Health-promoting physical activity of children who use Assistive Mobility devices: A scoping review. American Journal of Occupational Therapy, 70(5), 1-11. doi: 10.5014/ajot.2016.021543
Johns, DJ., Hartmann-Boyce, J., Jebb, SA., Aveyard, P. (2016). Weight change among people randomized to minimal intervention control groups in weight loss trials. *Obesity, 24*(4), 772-780. doi: 10.1002/oby.21255
Jones, M., Defever, E., Letsinger, A., Steele, J., & Mackintosh, KA. (2020). A mixed-studies systematic review and meta-analysis of school-based interventions to promote physical activity and/or reduce sedentary time in children. *Journal of Sport & Health Science, 9*(1), 3-17. doi: 10.1016/j.jshs.2019.06.009
Joseph, RP., & Maddock, JE. (2016). Observational park-based physical activity studies: A systematic review of the literature. Preventive Medicine, 89, 257-277. doi: 10.1016/j.ypmed.2016.06.016
Joseph, RP., Royse, KE., & Benitez, TJ. (2019). A systematic review of electronic and mobile health (E- and mHealth) physical activity interventions for African american and Hispanic women. *Journal of Physical Activity and Health, 16*(3), 230-239. 10.1123/jpah.2018-0103
Katigbak, C., Flaherty, E., Chao, Y., Nguyen, T., Cheung, D., & Kwan, RY. (2018). A systematic review of culturally specific interventions to increase physical activity for older Asian americans. Journal of cardiovascular Nursing, 33(4), 313-321. doi: 10.1097/JCN.0000000000000459
Kaushal, N., & Rhodes, RE. (2014). The home physical environment and its relationship with physical activity and sedentary behavior: A systematic review. *Preventive Medicine, 67*, 221-237. doi: 10.1016/j.ypmed.2014.07.026
Kelley, GA., Kelley, KS., & Pate, RR. (2014). Effects of exercise on BMI z-score in overweight and obese children and adolescents: A systematic review with meta-analysis. BMC Pediatrics, 14(1), 225. https://doi.org/10.1186/1471-2431-14-225
Kelley, GA., Kelley, KS., & Pate, RR. (2015). Exercise and BMI in overweight and obese children and adolescents: A systematic review and trial sequential meta-analysis. *BioMed Research International,* 2015, 704539. https://doi.org/10.1155/2015/704539
Keum, N., Ju, W., Lee, DH., Ding, EL., Hsieh, CC., Goodman, JE. … Giovannucci, EL. (2014). Leisure-time physical activity and endometrial cancer risk: Dose-response meta-analysis of epidemiological studies. *International Journal of Cancer, 135*(3), 682-694. doi: 10.1002/ijc.28687
Kong, A., Tussing-Humphreys, LM., Odoms-Young, AM., Stolley, MR. & Fitzgibbon, ML. (2014). Systematic review of behavioral interventions with culturally adapted strategies to improve diet and weight outcomes in African American women. *Obesity Reviews, 15*, 62-92. doi: 10.1111/obr.12203
Kopp, LM., Gastelum, Z., Guerrero, CH., Howe, CL., Hingorani, P., & Hingle, Me. (2017). Lifestyle behavior interventions delivered using technology in childhood, adolescent, and young adult cancer survivors: A systematic review. *Pediatric Blood & Cancer, 64*(1), 13-17. doi: 10.1002/pbc.26166
Kubacki, K., Ronti, R., Lahtinen, V., Pang, B., & Rundle-Thiele, S. (2017). Social marketing interventions aiming to increase physical activity among adults: A systematic review. *Health Education, 117*(1), 69-89. https://doi.org/10.1108/HE-02-2016-0008
Kumanyika, SK., Swank, M., Stachecki, J., Whitt-Glover, MC., & Brennan, LK. (2014). Examining the evidence for policy and environmental strategies to prevent childhood obesity in black communities: New directions and next steps. *Obesity Reviews, 15*, 177-203. doi: 10.1111/obr.12206
Kuzik, N., Poitras, VJ., Tremblay, MS., Lee, EY., Hunter, S., & Carson, V. (2017). Systematic review of the relationships between combinations of movement behaviours and health indicators in the early years (0-4 years). *BMC Public Health, 17*(5), 849. doi: 10.1186/s12889-017-4851-1
Lancaster, KJ., Carter-Edwards, L., Grilo, S, Shen, C., & Schoenthaler, AM. (2014). Obesity interventions in African American faith-based organizations: a systematic review. *Obesity Reviews, 15*(4), 159-176. doi: 10.1111/obr.12207
Larouche, R., Mammen, G., Rowe, DA., & Faulkner, G. (2018). Effectiveness of active school transport interventions: A systematic review and update. *BMC Public Health, 18*(1), 206. doi: 10.1186/s12889-017-5005-1
Lee, AM., Chavez, S., Bian, J., Thompson, LA., Gurka, MJ., Williamson, VG., & Modave, F. (2019). Efficacy and effectiveness of mobile health technologies for facilitating physical activity in adolescents: Scoping review. *JMIR mHealth and uHealth, 7*(2), e11847. https://dx.doi.org/10.2196/11847
Lee, Y., Yun, L., Kim, M., & Washington, M. (2018). A qualitative systematic review of public-private partnership in promoting physical activity. *Evaluation & the Health Professions, 43*(2), 90-104. doi: 10.1177/0163278718796153
Lemstra, M., Bird, Y., Nwankwo, C., Rogers, M., & Moraros, J. (2016). Weight loss intervention adherence and factors promoting adherence: a meta-analysis. Patient preference and adherence, 10, 1547-1559. doi: 10.2147/PPA.S103649
Levasseur, M., Généreux, M., Bruneau, J., Vanasse, A., Chabot, E., Beaulac, C., … Bédard, M. (2015). Importance of proximity to resources, social support, transportation and neighborhood security for mobility and social participation in older adults: Results from a scoping study. BMC Public Health, 15, 503. https://doi.org/10.1186/s12889-015-1824-0
Li, R., Qu, S., Zhang, P., Chattopadhyay, S., Gregg, EW., Albright, A., … Pronk, NP. (2015). Economic evaluation of combined diet and physical activity promotion programs to prevent type 2 diabetes among persons at increased risk: A systematic review for the community preventive services task force. *Annals of Internal Medicine, 163*(6), 452-460. doi: 10.7326/M15-0469
Liao, Y., Liao, J., Durand, CP., & Dunton, GF. (2014). Which type of sedentary behavior intervention is more effective at reducing body mass index in children? A meta-analytic review. *Obesity Reviews, 15*(3), 159-168. doi: 10.1111/obr.12112
Liao, Y., Skelton, K., Dunton, G., & Bruening, M. (2016). A systematic Review of methods and Procedures used in ecological Momentary assessments of diet and physical activity research in youth: An adapted STROBE checklist for reporting EMA studies (CREMAS). Journal of Medical internet research, 18(6), e151. doi: 10.2196/jmir.4954
Lin, X., Zhang, X., Guo, J., Roberts, CK., McKenzie, S., Wu, WC., … Song, Y. (2015). Effects of exercise training on cardiorespiratory fitness and biomarkers of cardiometabolic health: A systematic review and meta-analysis of randomized controlled trials. *Journal of the American Heart Association, 4*(7), 1-28 https://doi.org/10.1161/JAHA.115.002014
Ling, J., Robbins, LB., & Wen, F. (2016). Interventions to prevent and manage overweight or obesity in preschool children: A systematic review. *International Journal of Nursing Studies, 53*, 270-289. doi: 10.1016/j.ijnurstu.2015.10.017
Ling, J., Robbins, LB., Wen, F., & Peng, W. (2015). Interventions to increase physical activity in children aged 2-5 years: A systematic review. *Pediatric Exercise and Science, 27*(3), 314-333. 10.1123/pes.2014-0148
Liu, M., Wu, L., & Ming, Q. (2015). How does physical activity intervention improve self-esteem and self-concept in children and adolescents? Evidence from a meta-analysis. *PloS One, 10*(8), e0134804. doi: 10.1371/journal.pone.0134804
Loya, JC. (2018). Systematic review of physical activity interventions in Hispanic adults. *Hispanic Health Care International*, 16(4), 174-188. doi: 10.1177/1540415318809427
Lubans, D., Richards, J., Hillman, C., Faulkner, G., Beauchamp, M., Nilsson, M., … Biddle, S. (2016). Physical activity for cognitive and mental health in youth: A systematic review of mechanisms. *Pediatrics, 138*(3), e20161642; doi: https://doi.org/10.1542/peds.2016-1642
Ma, JK., & Martin Ginis, KA. (2018). A meta-analysis of physical activity interventions in people with physical disabilities: Content, characteristics, and effects on behavior. *Psychology of Sport and Exercise, 37*, 262-273. https://doi.org/10.1016/j.psychsport.2018.01.006
Macarthur, G., Caldwell, DM., Redmore, J., Watkins, SH., Kipping, R., White, J., . . . Campbell, R. (2018). Individual-, family-, and school-level interventions targeting multiple risk behaviours in young people. *Cochrane Database of Systematic Reviews*, 2018(10). 10.1002/14651858.CD009927.pub2
Mama, SK., McNeill, LH., McCurdy, SA., Evans, AE., Diamond, PM., Adamus-Leach, HJ., … Lee, RE. (2015). Psychosocial factors and theory in physical activity studies in minorities. *Am J Health Behav, 39*(1), 68-76. doi: 10.5993/AJHB.39.1.8
March, S., Torres, E., Ramos, M., Ripoll, J., Garcia, A., Bulilete, O., … Llobera, J. (2015). Adult community health-promoting interventions in primary health care: A systematic review. *Preventive Medicine,76*, S94-104. doi: 10.1016/j.ypmed.2015.01.016
Martin, R., & Murtagh, EM. (2017). Effect of active lessons on physical activity, academic, and health outcomes: A systematic review. *Research Quarterly for Exercise and Sport, 88*(2), 149-168. doi: 10.1080/02701367.2017.1294244
Maselli, M., Ward, PB., Gobbi, E., & Carraro, A. (2018). Promoting physical activity among university students: A systematic review of controlled trials. *American Journal of Health Promotion, 32*(7), 1602-1612. doi: 10.1177/0890117117753798
Mastellos, N., Gunn, LH., Felix, LM., Car, J., & Majeed, A. (2014). Transtheoretical model stages of change for dietary and physical exercise modification in weight loss management for overweight and obese adults. *Cochrane Database Syst Rev, 2*, CD008066. doi: 10.1002/14651858.CD008066.pub3
Mayne, SL., Auchincloss, AH., & Michael, YL. (2015). Impact of policy and built environment changes on obesity-related outcomes: A systematic review of naturally occurring experiments. *Obesity Reviews, 16*(5), 362-375. doi: 10.1111/obr.12269
McGarty, AM., Penpraze, V., & Melville, CA. (2014). Accelerometer use during field-based physical activity research in children and adolescents with intellectual disabilities: a systematic review. *Research Developmental Disabilities, 35*(5), 973-981. doi: 10.1016/j.ridd.2014.02.009
McGoey, T., Root, Z., Druner, M., & Barbi, L. (2016). Evaluation of physical activity interventions in children via the reach, efficacy/effectiveness, adoption, implementation, and maintenance (RE-AIM) framework: A systematic review of randomized and non-randomized trials. *Preventative Medicine*, 82, 8-9. doi: 10.1016/j.ypmed.2015.11.004
McGoey, T., Root, Z., Bruner, MW., & Law, B. (2015). Evaluation of physical activity interventions in youth via the reach, efficacy/Effectiveness, adoption, implementation, and maintenance (RE-AIM) framework: A systematic review of randomised and non-randomised trials. *Preventative Medicine*, 76:58-67. doi: 10.1016/j.ypmed.2015.04.006
McGrath, LJ., Hopkins, WG., & Hinckson, EA. (2015). Associations of objectively measured built-environment attributes with youth moderate-vigorous physical activity: A systematic review and meta-analysis. *Sports Medicine, 45*(6), 841-865. doi: 10.1007/s40279-015-0301-3
McKenzie, TL., & Smith, NJ. (2017). Studies of physical education in the United States using SOFIT: A review. *Research Quarterly Exercise and Sport, 88*(4), 492-502. doi: 10.1080/02701367.2017.1376028
McMichan, L., Gibson, AM., & Rowe, DA. (2018). Classroom-based physical activity and sedentary behavior interventions in adolescents: A systematic review and meta-analysis. *Journal of Physical Activity and Health*, 15(5):383-393. doi: 10.1123/jpah.2017-0087
McPherson, AC., Keith, R., & Swift, JA. (2014). Obesity prevention for children with physical disabilities: A scoping review of physical activity and nutrition interventions. Disability and Rehabilitation, 36(19), 1373-1587. doi: 10.3109/09638288.2013.863391
Melvin, CL., Jefferson, MS., Rice, LJ., Nemeth, LS., Wessell, AM., Nietert, PJ. … Hughes-Halbert, C. (2017). A systematic review of lifestyle counseling for diverse patients in primary care. *Preventive Medicine, 100*, 67-75. doi: 10.1016/j.ypmed.2017.03.020
Mendonça, G., Cheng, LA., Mélo, EN., & de Farias, JC. (2014). Physical activity and social support in adolescents: A systematic review. *Health Education Research, 29*(5), 822-839. doi: 10.1093/her/cyu017
Meng, L., Wolff, MB., Mattick, KA., DeJoy, DM., Wilson, MG. & Smith, ML. Strategies for worksite health interventions to employees with elevated risk of chronic diseases. Safety and health at work, 8(2), 117-129. doi: 10.1016/j.shaw.2016.11.004
Miller, KJ., Adair, BS., Pearce, AJ., said, CM., Ozanne, E., & Morris, MM. (2014). Effectiveness and feasibility of virtual reality and gaming system use at home by older adults for enabling physical activity to improve health-related domains: A systematic review. *Age and Ageing, 43*(2), 188-195. doi: 10.1093/ageing/aft194
Millstein, RA. (2014). Measuring outcomes in adult weight loss studies that include diet and physical activity: a systematic review. *Journal of Nutrition and Metabolism,* 2014(421423), 1-13. https://doi.org/10.1155/2014/421423
Mita, G., Mhurchu CN., & Jull, A. (2016). Effectiveness of social media in reducing risk factors for noncommunicable diseases: a systematic review and meta-analysis of randomized controlled trials. Nutrition reviews, 74(4), 237-247. doi: 10.1093/nutrit/nuv106
Molema, CCM., Wendel-Vos, GCW, Puijk, L., Jensen, JD., Schuit, J., & de Wit, GA. (2016). A systematic review of financial incentives given in the healthcare setting; do they effectively improve physical activity levels? *BMC Sports Science, Medicine & Rehabilitation, 8*, 15. doi: 10.1186/s13102-016-0041-1
Moore, CC., McCullough, AK., Aguiar, EJ., Ducharme, SW., & Tudor-Locke, C. (2020). Toward Harmonized Treadmill-based Validation of Step-Counting Wearable Technologies: A scoping Review. *Journal of Physical Activity and Health,* 2020, 1-13. https://dx.doi.org/10.1123/jpah.2019-0205
Moredich, CA. & Kessler, TA. (2014). Physical activity and Nutritional weight loss interventions in obese, low-income women: An integrative review. Journal of Midwifery and Women’s health, 59(4), 380-387. doi: 10.1111/jmwh.12061
Morgan, EH., Schoonees, A., Sriram, U., Faure, M., & Seguin-Fowler, RA. (2020). Caregiver involvement in interventions for improving children’s dietary intake and physical activity behaviors. *Cochrane database of systematic reviews, 2020*(1), CD012547. https://dx.doi.org/10.1002/14651858.CD012547.pub2
Morgan, PJ., Young, MD., Lloyd, AB., Wang, ML., Eather, N., Miller, A. … Pagoto, SL. (2017). Involvement of fathers in pediatric obesity treatment and prevention trials: A systematic review. *Pediatrics, 139*(2), e20162635. doi: 10.1542/peds.2016-2635
Mosdøl, A., Lidal, IB., Straumann, GH., & Vist, GE. (2017). Targeted mass media interventions promoting healthy behaviours to reduce risk of non-communicable diseases in adult, ethnic minorities. *Cochrane Database of Systematic Reviews, 2*(2):CD011683. doi: 10.1002/14651858.CD011683.pub2
Mudaliar, U., Zabetian, A., Goodman, M., Echouffo-Tcheugui, JB., Albright, AL., Gregg, EW. … Ali, MK. (2016). Cardiometabolic risk factor changes observed in diabetes prevention programs in US settings: A systematic review and meta-analysis. *PLoS Medicine, 13*(7), e1002095. https://doi.org/10.1371/journal.pmed.1002095
Muir, SD., Silva, SSM., Woldegiorgis, MA., Rider, H., Meyer, D., & Jayawardana, MW. (2019). Predictors of success of workplace physical activity interventions: A systematic review. *Journal of Physical Activity & Health, 16*(8), 647-656. https://dx.doi.org/10.1123/jpah.2018-0077
Mutschler, C., Naccarato, E., Rouse, J., Davey, C., & McShane, K. (2018). Realist-informed review of motivational interviewing for adolescent health behaviors. *Systematic Reviews*, 7(1), 109-121. doi:10.1186/s13643-018-0767-9
Nathan, N., Elton, B., Babic, M., McCarthy, N., Sutherland, R., Presseau, J., … Wolfenden, L. (2018). Barriers and facilitators to the implementation of physical activity policies in schools: A systematic review. *Preventive Medicine, 107*, 45-53. https://doi.org/10.1016/j.ypmed.2017.11.012
Naylor, PJ., Nettlefold, L., Race, D., Hoy, C., Ashe, MC., Wharf, H., … McKay, H. (2015). Implementation of school based physical activity interventions: a systematic review. *Preventative Medicine, 72*, 95-115. doi: 10.1016/j.ypmed.2014.12.034
Newton Jr, RL., Griffith, DM., Kearney, WB., & Bennett, GG. (2014). A systematic review of weight loss, physical activity and dietary interventions involving African american men. *Obesity Reviews, 15*(Supp 4), 93-106. doi: 10.1111/obr.12209
Ng, MK., Yousuf, B., Bigelowa, PL., Van Eerd, D. (2015). Effectiveness of health promotion programmes for truck drivers: A systematic review. *Health Education Journal, 74*(3), 270-286. https://doi.org/10.1177/0017896914533953
Olanrewaju, O., Kelly, S., Cowan, A., Brayne, C., & Lafortune, L. (2016). Physical activity in community dwelling older people: A systematic review of reviews of interventions and context. *PloS One, 11*(12), e0168614. doi: 10.1371/journal.pone.0168614
Olstad, DL., Ancilotto, R., Teychenne, M., Minaker, LM., Taber, DR., Raine, KD. … Ball, K. (2017). Can targeted policies reduce obesity and improve obesity-related behaviours in socioeconomically disadvantaged populations? A systematic review. *Obesity Reviews, 18*(7), 791-807. doi: 10.1111/obr.12546
Olstad, DL, Teychenne, M., Minaker, LM., Taber, DR., Raine, KD., Nykiforuk, CIJ., … Ball, K. (2016). Can policy ameliorate socioeconomic inequities in obesity and obesity-related behaviours? A systematic review of the impact of universal policies on adults and children. *Obesity Reviews, 17*(12):1198-1217. doi: 10.1111/obr.12457
Olstad, DL., Campbell, EJ., Raine, KD., & Nykiforuk, CIJ. (2015). A multiple case history and systematic review of adoption, diffusion, implementation and impact of provincial daily physical activity policies in canadian schools. *BMC Public Health*, 15, 385-410. doi: 10.1186/s12889-015-1669-6
Orr, K., Evans, MB., Tamminen, KA., & Arbour-Nicitopoulos, KP. (2020). A scoping review of recreational sport programs for disabled emerging adults. *Research Quarterly for Exercise & Sport, 91*(1), 142-157. https://doi.org/10.1080/02701367.2019.1653432
Patnode, CD., Evans, CV., Senger, CA., Redmond, N., & Lin, JS. (2017). Behavioral counseling to promote a healthful diet and physical activity for cardiovascular disease prevention in adults without known cardiovascular disease risk factors: Updated evidence report and systematic review for the US preventive services task force. *JAMA, 318*(2), 175-193. doi: 10.1001/jama.2017.3303
Peaceman, AM., Clifton, RG., Phelan, S., Gallagher, D., Evans, M., Redman, LM., … Cahill, AG. (2018). Lifestyle interventions limit Gestational weight gain in women with overweight or obesity: LIFE-Moms prospective meta-analysis. *Obesity, 26*(9), 1396-1404. doi: 10.1002/oby.22250
Pearson, N., Braithwaite, R., & Biddle, SJH. (2015). The effectiveness of interventions to increase physical activity among adolescent girls: A meta-analysis. *Academic Pediatrics, 15*(1), 9-18. doi: 10.1016/j.acap.2014.08.009
Pelletier, CA., Smith-Forrester, J., & Klassen-Ross, T. (2017). A systematic review of physical activity interventions to improve physical fitness and health outcomes among indigenous adults living in Canada. *Preventive Medicine Reports*, 8, 242-249. doi: 10.1016/j.pmedr.2017.11.002
Pescatello, LS., Buchner, DM., Jakicic, JM., Powell, KE., Kraus, WE., Bloodgood, B., . . . Piercy, KL. (2019). Physical activity to prevent and treat hypertension: A systematic review. *Medicine and Science in Sports and Exercise, 51*(6), 1314-1323. 10.1249/MSS.0000000000001943
Petrescu-Prahova, MG., Eagen, TJ., Fishleder, SL., & Belza, B. (2017). Enhance ® fitness dissemination and implementation,: 2010-2015: A scoping review. *American Journal of Preventative Medicine*, 52(3), S295-S299. doi: 10.1016/j.amepre.2016.08.015
Pitchford, EA., Dixon-Ibarra, A., & Hauck, JL. (2018). Physical activity research in intellectual disability: A scoping review using the behavioral epidemiological framework. *American Journal on Intellectual and Developmental Disabilities, 123*(2), 140-163. doi: 10.1352/1944-7558-123.2.140
Poitras, VJ., Gray, CE., Borghese, MM., Carson, V., Chaput, J., Janssen, I., …Tremblay, MS. (2016). Systematic review of the relationships between objectively measured physical activity and health indicators in school-aged children and youth. *Applied Physiology, Nutrition, and Metabolism, 41*(6), S197-239. doi: 10.1139/apnm-2015-0663
Rajjo, T., Mohammed, K., Alsawas, M., Ahmed, AT., Farah, W., Asi, N., … Murad, MH. Treatment of pediatric obesity: An umbrella systematic review. *Journal of Clinical Endocrinology and Metabolism, 102*(3), 763-775. doi: 10.1210/jc.2016-2574
Ramchand, R., Ahluwalia, SC., Xenakis, L., Apaydin, E., Raaen, L., & Grimm, G. (2017). A systematic review of peer-supported interventions for health promotion and disease prevention. *Preventive Medicine, 101*, 156-170. doi: 10.1016/j.ypmed.2017.06.008
Rathore, A., & Lom, B. (2017). The effects of chronic and acute physical activity on working memory performance in healthy participants: A systematic review with meta-analysis of randomized controlled trials. *Systematic Reviews, 6*(1), 124. doi: 10.1186/s13643-017-0514-7
Reed, JL., Prince, SA., Elliott, CG., Mullen, KA., Tulloch, HE., Hiremath, S., … Reid, RD. (2017). Impact of workplace physical activity interventions on physical activity and cardiometabolic health among working-age women. *Circulation: Cardiovascular Quality and Outcomes, 10*(2), e003516. doi: 10.1161/CIRCOUTCOMES.116.003516
Reed, M., Wilbur, J., & Schoeny, M. (2015). Parent and African American Daughter obesity prevention interventions: An integrative review. *Journal of Health Care for the Poor and Underserved, 26*(3), 737-760. doi: 10.1353/hpu.2015.0103
Rhodes, RE., Baranova, M., Christian, H., & Westgarth, C. (2020). Increasing physical activity by four legs rather than two: Systematic review of dog-facilitated physical activity interventions. *British Journal of Sports Medicine, 54*, 1202-1207. 10.1136/bjsports-2019-101156
Richards, EA., & Cai, Y. (2016). Integrative review of nurse-delivered community-based physical activity promotion. *Applied Nursing Research, 31*, 132-138. doi: 10.1016/j.apnr.2016.02.004
Richards, EA., & Cai, Y. (2016). Integrative review of nurse-delivered physical activity interventions in primary care. *Western Journal of Nursing Research, 38*(4):484-507. doi: 10.1177/0193945915581861
Richards, EA. & Cai, Y. (2016). Physical activity outcomes of nurse-delivered lifestyle interventions. *Home Healthcare Now, 34*(2), 93-101. doi: 10.1097/NHH.0000000000000334
Ridgers, ND., McNarry, MA., & Mackintosh, KA. (2016). Feasibility and effectiveness of using wearable activity trackers in youth: A systematic review. *JMIR mHealth and uHealth, 4*(4), e129. doi: 10.2196/mhealth.6540
Robinson, MN., Tansil, KA., Elder, RW., Soler, RE., Labre, MP., Mercer, SL., … Rimer BK. (2014). Mass media health communication campaigns combined with health-related product distribution: A community guide systematic review. *American Journal of Preventative Medicine, 47*(3), 360-371. doi: 10.1016/j.amepre.2014.05.034
Romain, AJ., Bernard, P., Akrass, Z., St-Amour, S., Lachance, J. P., Hains-Monfette, G., . . . Abdel-Baki, A. (2020). Motivational theory-based interventions on health of people with several mental illness: A systematic review and meta-analysis. *Schizophrenia Research, 222*, 31-41.. doi: 10.1016/j.schres.2020.05.049
Rosen, L, French, A., & Sullivan, G. (2015). Complementary, holistic, and integrative medicine: Yoga. *Pediatrics in Review, 36*(10), 468-474. doi: 10.1542/pir.36-10-468
Rossi, A., Friel, C., Carter, L., & Garber, CE. (2018). Effects of theory-based behavioral interventions on physical activity among overweight and obese female cancer survivors: A systematic review of randomized controlled trials. *Integrated Cancer Therapies*, 17(2), 226-236. doi: 10.1177/1534735417734911
Schembre, SM., Liao, Y., Robertson, MC., Dunton, GF., Kerr, J., Meghan, E., … Hicklen RS. (2018). Just-in-time feedback in diet and physical activity interventions: Systematic review and practical design framework*. Journal of Medical Internet Research, 20*(3), e106. doi: 10.2196/jmir.8701
Schuch, FB., Vancampfort, D., Sui, X., Rosenbaum, S., Firth, J., Richards, J., … Stubbs, B. (2016). Are lower levels of cardiorespiratory fitness associated with incident depression? A systematic review of prospective cohort studies. *Preventive Medicine, 93*, 159-165. https://doi.org/10.1016/j.ypmed.2016.10.011
Shirley, K., Rutfield, R., Hall, N., Fedor, N., McCaughey, VK., & Zajac, K. (2014). Combinations of obesity prevention strategies in US elementary schools: A critical review. *Journal of Primary Prevention, 36*(1), 1-20. doi: 10.1007/s10935-014-0370-3
Silva, SSM., Jayawardana, MW., & Meyer, D. (2018). Statistical methods to model and evaluate physical activity programs, using step counts: A systematic review. *PloS One, 13*(11), e0206763. https://dx.doi.org/10.1371/journal.pone.0206763
Sims, J., Scarborough, P., & Foster, C. (2015). The effectiveness of interventions on sustained childhood physical activity: A systematic review and meta-analysis of controlled studies. *PloS One, 10*(7), e0132935. doi: 10.1371/journal.pone.0132935
Sirois, FM., Kitner, R., & Hirsch, JK. (2015). Self-compassion, affect, and health-promoting behaviors. *Health psychology, 34*(6), 661-669. doi: 10.1037/hea0000158
Sisson, SB., Krampe, M., Anundson, K., & Castle, S. (2016). Obesity prevention and obesogenic behavior interventions in child care: A systematic review. *Preventative Medicine, 87*, 57-69. doi: 10.1016/j.ypmed.2016.02.016
Specker, B., Thiex, NW., & Sudhagoni, RG. (2015). Does exercise influence pediatric bone? A systematic review. *Clinical Orthopaedics and Related Research. 473*(11), 3658-3672. doi: 10.1007/s11999-015-4467-7
Stanhope, KK., Kay, C., Stevenson, B., & Gazmararian, JA. (2017). Measurement of obesity prevention in childcare settings: A systematic review of current instruments. *Obesity Research & Clinical Practice*, 11(5), 52-89. doi: 10.1016/j.orcp.2016.06.002.edwards
Stasi, S., Spengler, J., Maddock, J., McKyer, L., & Clark, H. (2019). Increasing access to physical activity within low income and diverse communities: A systematic review. *American Journal of Health Promotion, 33*(6), 933-940. https://dx.doi.org/10.1177/0890117119832257
Street, TD., Lacey, SJ., & Langdon, RR. (2017). Gaming your way to health: A systematic review of exergaming programs to increase health and exercise behaviors in adults. *Games Health J, 6*(3), 136-146. doi: 10.1089/g4h.2016.0102
Strohacker, K., Fazzino, D., Breslin, WL., & Xu, X. (2015). The use of periodization in exercise prescriptions for inactive adults: A systematic review. *Preventive Medicine Reports, 2*, 385-396. doi: 10.1016/j.pmedr.2015.04.023
Stuckey, MI., Carter, SW., & Knight, E. (2017). The role of smartphones in encouraging physical activity in adults. *International Journal of General Medicine, 10*, 293-303. doi: 10.2147/IJGM.S134095
Sypes, EE., Newton, G., & Lewis, ZH. (2019). Investigating the use of an electronic activity monitor system as a component of physical activity and weight-loss interventions in nonclinical populations: A systematic review. *Journal of Physical Activity & Health, 16*(4), 294-302. https://dx.doi.org/10.1123/jpah.2017-0660
Szuhany, KL., Bugatti, M., & Otto, MW. (2015). A meta-analytic review of the effects of exercise on brain-derived neurotrophic factor. *Journal of Psychiatric Research, 60,* 56-64. doi: 10.1016/j.jpsychires.2014.10.003
Temple, VA., Frey, GC., & Stanish, HI. (2017). Interventions to promote physical activity for adults with intellectual disabilities. *Salud Publica de Mexico, 59*(4), 446-453. doi: 10.21149/8218
Towns, C., Cooke, M., Rysdale, L., & Wilk, P. (2014). Healthy weights interventions in aboriginal children and youth: A review of the literature. Can J Diet Pract and Res, 75(3), 125-131. https://doi.org/10.3148/cjdpr-2014-006
Parra, MT., Portírio, GJM., Arredondo, EM., & Atallah, ÁN. (2018). Physical activity interventions in faith-based organizations: A systematic review. *Am J Health Promot, 32*(3), 677-690. doi: 10.1177/0890117116688107
Vazou, S., Webster, CA., Stewart, G., Candal, P., Egan, CA., Pennell, A., … Russ, LB. (2020). A systematic review and qualitative synthesis resulting in a typology of elementary classroom movement Integration interventions. *Sports Medicine - Open, 6*(1). 10.1186/s40798-019-0218-8
Verjans-Janssen, SRB., van de Kolk, I., Van Kann, DHH., Kremers, SPJ., & Gerards, SMPL. (2018). Effectiveness of school-based physical activity and nutrition interventions with direct parental involvement on children’s BMI and energy balance-related behaviors - A systematic review. *PloS one, 13*(9), e0204560. doi: 10.1371/journal.pone.0204560
Villa-González, E., Barranco-Ruiz, Y., Evenson, K. R., & Chillón, P. (2018). Systematic review of interventions for promoting active school transport. Preventive medicine, 111, 115-134. 10.1016/j.ypmed.2018.02.010
Wang, Y., Cai, L., Wu, Y., Wilson, RF., Weston, C., Fawole, O., … Segal, J. (2015). What childhood obesity prevention programmes work? A systematic review and meta-analysis. *Obesity Reviews, 16*(7), 547-565. doi: 10.1111/obr.12277
Warburton, DER., & Bredin, SSD. (2017). Health benefits of physical activity: A systematic review of current systematic reviews. *Current Opinion in Cardiology, 32*(5), 541-556. doi: 10.1097/HCO.0000000000000437
Ward, DS., Welker, E., Choate, A., Henderson, KE., Lott, M., Tovar, A. … Sallis, JF. (2017). Strength of obesity prevention interventions in early care and education settings: A systematic review. *Preventive Medicine, 95*, S37-S52. doi: 10.1016/j.ypmed.2016.09.033
Weatherson, KA., Gainforth, HL., & Jung, ME. (2017). A theoretical analysis of the barriers and facilitators to the implementation of school-based physical activity policies in Canada: A mixed methods scoping review. *Implement Science*, 12(1), 41-56. doi: 10.1186/s13012-017-0570-3
Wiese, CW., Kuykendall, L., & Tay, L. (2018). Get active? A meta-analysis of leisure-time physical activity and subjective well-being. *Journal of Positive Psychology, 13*(1), 57-66. https://doi.org/10.1080/17439760.2017.1374436
Williams, G., Hamm, MP., Shulhan, J., Vandermeer, B., & Hartling, L. (2014). Social media interventions for diet and exercise behaviours: A systematic review and meta-analysis of randomised controlled trials. *BMJ Open, 4*(2), e003926. doi:10.1136/bmjopen-2013-003926
Wilson, K., Senay, I., Durantini, M., Sanchez, F., Hennessy, M., Spring, B., … Albarracin, D. (2015). When it comes to lifestyle recommendations, more is sometimes less: a meta-analysis of theoretical assumptions underlying the effectiveness of interventions promoting multiple behavior domain change. *Psychological Bulletin, 141*(2), 474-509. doi: 10.1037/a0038295
Winter, SJ., Sheats, JL., & King, AC. (2016). The use of behavior change techniques and theory in technologies for cardiovascular disease prevention and treatment in adults: A comprehensive review. *Progress in Cardiovascular Disease*, 58(6), 605-612. doi: 10.1016/j.pcad.2016.02.005
Wolfenden, L., Jones, J., Williams, CM., Finch, M., Wyse, RJ., Kingsland, M., … Yoong, SL. (2016). Strategies to improve the implementation of healthy eating, physical activity and obesity prevention policies, practices or programmes within childcare services. *Cochrane Database of Systematic Reviews*. 2016;10:
Wolfenden, L., Barnes, C., Jones, J., Finch, M., Wyse, R. J., Kingsland, M., . . . Yoong, S. L. (2020). Strategies to improve the implementation of healthy eating, physical activity and obesity prevention policies, practices or programmes within childcare services. *Cochrane Database of Systematic Reviews, 2020*(2), 011779. https://dx.doi.org/10.1002/14651858.CD011779.pub3
Wolfenden, L., Goldman, S., Stacey, FG., Grady, A., Kingsland, M., Williams, CM., . . . Yoong, SL. (2018). Strategies to improve the implementation of workplace-based policies or practices targeting tobacco, alcohol, diet, physical activity and obesity. *Cochrane Database of Systematic Reviews*, 2018(11), 012439. 10.1002/14651858.CD012439.pub2
Won, J., Lee, C., Forjuoh, SN., & Ory, MG. (2016). Neighborhood safety factors associated with older adults' health-related outcomes: A systematic literature review. *Social Science Medicine, 165*, 177-186. doi: 10.1016/j.socscimed.2016.07.024
Wu, Y., MacDonald, HV., & Pescatello, LS. (2016). Evaluating exercise prescription and instructional methods used in tai chi studies aimed at improving balance in older adults: A systematic review. *Journal of the American Geriatrics Society, 64*(10), 2074-2080. doi: 10.1111/jgs.14242
Xia, Y., Deshpande, S., & Bonates, T. (2016). Effectiveness of social marketing interventions to promote physical activity among adults: A systematic review. *Journal of Physical Activity & Health, 13*(11), 1263-1274. doi: 10.1123/jpah.2015-0189
Yen, IH., Flood, JF., Thompson, H, Anderson, LA, & Wong, G. (2014). How design of places promotes or inhibits mobility of older adults: Realist synthesis of 20 years of research. *Journal of Aging Health, 26*(8), 1340-1372. doi: 10.1177/0898264314527610
Yun, L., Ori, EM., Lee, Y., Sivak, A., & Berry, TR. (2017). A systematic review of community-wide media physical activity campaigns: An update from 2010. Journal of physical activity and health, 14(7):552-570. doi: 10.1123/jpah.2016-0616
Zubala, A., MacGillivray, S., Frost, H., Kroll, T., Shelton, DA., Gavine, A., … Morris, J. (2017). Promotion of physical activity interventions for community dwelling older adults: A systematic review of reviews. *PloS One, 12*(7), e0180902. doi: 10.1371/journal.pone.0180902

**Table A3.** Preferred Reporting Items for Systematic Reviews and Meta-Analyses Extension for Scoping Reviews (PRISMA-ScR) Checklist.

Section	Item	PRISMA-ScR checklist Item	Reported on Page #
Title			
Title	1	Identify the report as a scoping review	Title page
Abstract			
Structured summary	2	Provide a structured summary that includes (as applicable): Background, objectives, eligibility criteria, sources of evidence, charting methods, results, and conclusions that relate to the review questions and objectives	Page 2
Introduction			
Rationale	3	Describe the rationale for the review in the context of what is already known. Explain why the review questions/objectives lend themselves to a scoping review approach	Page 3–8
Objectives	4	Provide an explicit statement of the questions and objectives being addressed with reference to their key elements (e.g., population or participants, concepts, and context) or other relevant key elements used to conceptualize the review questions and/or objectives	Page 7–8
Methods			
Protocol and registration	5	Indicate whether a review protocol exists; state if and where it can be accessed (e.g., a Web address); and if available, provide registration information, including the registration number	Page 8
Eligibility criteria	6	Specify characteristics of the sources of evidence used as eligibility criteria (e.g., years considered, language, and publication status), and provide a rationale	Appendix A
Information sources^a^	7	Describe all information sources in the search (e.g., databases with dates of coverage and contact with authors to identify additional sources), as well as the date the most recent search was executed	Page 8–9
Search	8	Present the full electronic search strategy for at least one database, including any limits used, such that it could be repeated	Appendix A
Selection of sources of evidence^b^	9	State the process for selecting sources of evidence (i.e., screening and eligibility) included in the scoping review	Page 8–9
Data charting process^c^	10	Describe the methods of charting data from the included sources of evidence (e.g., calibrated forms or forms that have been tested by the team before their use, and whether data charting was done independently or in duplicate) and any processes for obtaining and confirming data from investigators	Page 9
Data items	11	List and define all variables for which data were sought and any assumptions and simplifications made	Page 9
Critical appraisal of individual sources of evidence^d^	12	If done, provide a rationale for conducting a critical appraisal of included sources of evidence; describe the methods used and how this information was used in any data synthesis (if appropriate)	N/A
Synthesis of results	13	Describe the methods of handling and summarizing the data that were charted	Page 9
Results			
Selection of sources of evidence	14	Give numbers of sources of evidence screened, assessed for eligibility, and included in the review, with reasons for exclusions at each stage, ideally using a flow diagram	Figure 1
Characteristics of sources of evidence	15	For each source of evidence, present characteristics for which data were charted and provide the citations	Page 10–18
Critical appraisal within sources of evidence	16	If done, present data on critical appraisal of included sources of evidence (see item 12)	N/A
Results of individual sources of evidence	17	For each included source of evidence, present the relevant data that were charted that relate to the review questions and objectives	Page 10–18
Synthesis of results	18	Summarize and/or present the charting results as they relate to the review questions and objectives	Page 10–18
Discussion			
Summary of evidence	19	Summarize the main results (including an overview of concepts, themes, and types of evidence available), link to the review questions and objectives, and consider the relevance to key groups	Page 18–21
Limitations	20	Discuss the limitations of the scoping review process	Page 22
Conclusions	21	Provide a general interpretation of the results with respect to the review questions and objectives, as well as potential implications and/or next steps	Page 22–23
Funding			
Funding	22	Describe sources of funding for the included sources of evidence, as well as sources of funding for the scoping review. Describe the role of the funders of the scoping review	Title page

JBI = Joanna Briggs Institute; PRISMA-ScR = Preferred Reporting Items for Systematic reviews and Meta-Analyses extension for Scoping Reviews. From: Ref.^22^

^a^Where *sources of evidence* (see second footnote) are compiled from, such as bibliographic databases, social media platforms, and Web sites.

^b^A more inclusive/heterogeneous term used to account for the different types of evidence or data sources (e.g., quantitative and/or qualitative research, expert opinion, and policy documents) that may be eligible in a scoping review as opposed to only studies. This is not to be confused with *information sources* (see first footnote).

^c^The frameworks by Arksey and O’Malley^20^ (6) and Levac and colleagues^21^ (7) and the JBI guidance (4, 5) refer to the process of data extraction in a scoping review as data charting*.*

^d^The process of systematically examining research evidence to assess its validity, results, and relevance before using it to inform a decision. This term is used for items 12 and 19 instead of “risk of bias” (which is more applicable to systematic reviews of interventions) to include and acknowledge the various sources of evidence that may be used in a scoping review (e.g., quantitative and/or qualitative research, expert opinion, and policy document).
